# Natural products targeting the immune-metabolic regulatory network in pulmonary hypertension: mechanisms, classification, and therapeutic prospects

**DOI:** 10.3389/fphar.2025.1712903

**Published:** 2026-01-08

**Authors:** Jianli Ma, Xiyu Cao, Xiuli Yang, Yi Zhu, Hualing Wang, Yundou Liu, Ke Chen, Xiao Liu, Chuantao Zhang

**Affiliations:** 1 Department of Respiratory Medicine, Hospital of Chengdu University of Traditional Chinese Medicine, Chengdu, Sichuan, China; 2 Chengdu Xinjin District Hospital of Traditional Chinese Medicine, Chengdu, Sichuan, China; 3 Department of Respiratory and Critical Care Medicine, Affiliated Fifth People’s Hospital of Chengdu University of Traditional Chinese Medicine, Chengdu, Sichuan, China

**Keywords:** pulmonary hypertension, natural products, immuno-metabolic crosstalk, vascular remodeling, metabolic reprogramming

## Abstract

Pulmonary hypertension (PH) is a clinical syndrome characterized by progressive elevation of pulmonary vascular resistance, whose pathological process is closely related to the crosstalk between immune dysregulation and metabolic remodeling, forming a complex immune-metabolic regulatory network. The abnormal activation and polarization of innate immune cells, as well as the imbalance and autoreactivity of the adaptive immune system, jointly drive the pulmonary vascular inflammatory response. Metabolic reprogramming of pulmonary artery smooth muscle cells, pulmonary artery endothelial cells, and others provides the energy and material basis for abnormal proliferation of vascular cells. Abnormal products generated by metabolic disorders can regulate energy metabolism of immune cells and inflammatory signals; abnormal activation of immune signals can remodel metabolic pathways. The two interact through mechanisms such as the mitochondria-inflammasome axis and epigenetic regulation, collectively promoting vascular remodeling. Targeting this network, natural products exhibit unique advantages due to their characteristics of multiple components and multiple targets. Among them, immunomodulatory natural products can improve the immune microenvironment by inhibiting core inflammatory pathways and regulating immune cell infiltration. Metabolic regulatory natural products focus on restoring mitochondrial function and correcting abnormal glucose and lipid metabolism to interfere with metabolic remodeling. By targeting mTOR, SIRT1 and other pivotal molecules, immune metabolic dual regulatory natural products can synchronously regulate immune response and metabolic activities, and block the vicious cycle of immune activation, metabolic disorder and vascular remodeling. However, challenges remain, including insufficient mechanistic depth and a significant gap between preclinical models and clinical application. Further research into these mechanisms may provide novel insights for PH therapy and improve patient prognosis.

## Graphical Abstract

**Figure:**
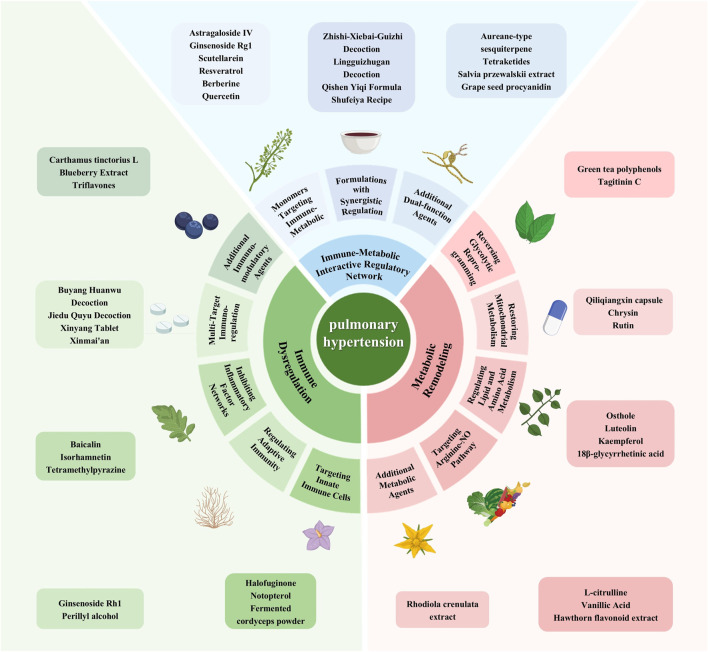

## 1 Introduction

Pulmonary hypertension (PH) is a clinical syndrome characterized by progressive elevation of pulmonary vascular resistance (PVR), defined as a mean pulmonary artery pressure >20 mmHg at rest. Among them, pulmonary arterial hypertension (PAH, WHO Group 1) requires meeting the criteria of PVR >2 Wood units and pulmonary artery wedge pressure (PAWP) ≤15 mmHg (Olsson et al., 2023; Kovacs et al., 2024). Its pathological features include intimal fibrosis, medial hypertrophy, and vascular remodeling of small pulmonary arteries, eventually leading to right ventricular hypertrophy and failure (Olsson et al., 2023). The clinical classification follows the WHO categorization, covering PAH, PH associated with left heart disease (Group 2), PH associated with lung diseases/hypoxia (Group 3), chronic thromboembolic pulmonary hypertension (Group 4), and PH with unclear mechanisms (Group 5) (Olsson et al., 2023; Weatherald et al., 2024). Epidemiological data show that the age-standardized incidence rate (ASIR) of global PAH in 2021 was 0.52 per 100,000 population, and the age-standardized prevalence rate was 2.28 per 100,000 population. The burden is significantly higher in women than in men, with marked regional disparities (Huang et al., 2025; Leary et al., 2025; Liu et al., 2025). Although the mortality rate and disability-adjusted life years showed a decreasing trend from 1990 to 2021, the incidence and prevalence continue to rise due to population growth and aging, and the ASIR is projected to increase to 0.57 per 100,000 population by 2050 (Huang et al., 2025; Leary et al., 2025; Liu et al., 2025).

Traditional treatment methods face multiple challenges. Targeted drugs such as endothelin receptor antagonists and prostacyclin analogs can improve symptoms, but their high cost and limited reimbursement restrict accessibility in low-resource areas (Nathan, 2023; Olsson et al., 2023). The treatment of PAH-related heart failure still mainly focuses on symptomatic management, lacking effective means to reverse the cardiopulmonary interactive damage (Leary et al., 2025; Liu et al., 2025). In addition, the response to targeted drugs varies significantly among some patients, which may be related to the irreversibility of vascular remodeling or comorbidities. End-stage patients have to rely on lung transplantation or ventricular assist devices, further highlighting the limitations of current treatments (Olsson et al., 2023). Therefore, exploring new therapeutic strategies and targets has become an urgent need in the field of PH research.

Recent studies have indicated that the occurrence and progression of PH are closely associated with the interaction between immune dysregulation and metabolic remodeling, forming a complex immune-metabolic regulatory network. The abnormal activation and polarization of innate immune cells, such as macrophages and neutrophils, and the imbalance and self-reactivity of adaptive immune system, such as B lymphocytes (B cells) and T lymphocytes (T cells), jointly drive the inflammatory response of pulmonary vessels (Hu et al., 2020; Scott et al., 2021; Liu S.-F. et al., 2022; Ni et al., 2022; Zhang Y. et al., 2024). Metabolic remodeling in pulmonary artery smooth muscle cells (PASMCs) and pulmonary artery endothelial cells (PAECs), including enhanced glycolysis and mitochondrial dysfunction, provides the energy and material basis for abnormal proliferation of vascular cells (Boehme et al., 2016; Li D. et al., 2021; Colon Hidalgo et al., 2022; Xu et al., 2024; Li et al., 2025). Abnormal metabolites under metabolic disorders drive the vascular inflammatory microenvironment by regulating energy metabolism and inflammatory signals in immune cells (Plecitá-Hlavatá et al., 2017; Hudalla et al., 2019; Hashimoto and Gupte, 2022). Aberrant activation of immune signals can reshape metabolic pathways such as glycolysis and fatty acid oxidation, and the mitochondria-inflammasome axis releases mitochondrial DNA (mtDNA), further amplifying inflammatory responses (D'Alessandro et al., 2018; Foley et al., 2022; Mavrogiannis et al., 2022). This multi-dimensional interaction involves metabolic reprogramming, immune microenvironment, organelle crosstalk, and epigenetic regulation of gene expression, ultimately promoting vascular remodeling and collectively driving the pathological process of PH (Chen et al., 2017; Ranasinghe et al., 2023).

Against this backdrop, natural products have shown unique advantages in regulating complex pathological networks due to their characteristics of multi-component, multi-target, low toxicity, and good biocompatibility (Ho et al., 2018). Natural products refer to biologically active compounds extracted from organisms such as plants, animals, and microorganisms. Their structural diversity and biocompatibility make them important resources for drug discovery, capable of interfering with the immune-metabolic network of PH through multiple pathways (Zhang et al., 2021; Najmi et al., 2022; Yu et al., 2022). Current research has confirmed that various natural products, including flavonoids, alkaloids, polyphenols, terpenoids, and polysaccharides, can improve hemodynamics and vascular remodeling in PH animal models, with mechanisms involving the regulation of signaling axes such as HIF-1α, mTOR, and AMPK (Zhang et al., 2021; Cui et al., 2022; Yu et al., 2022; Zhang J.-j. et al., 2024).

This review aims to systematically sort out the core mechanisms of the immune-metabolic interactive regulatory network in pulmonary hypertension, and focus on commenting on natural products targeting this network, including the action characteristics, molecular mechanisms and research progress of monomers, compound formulas and extracts. By in-depth analysis of the advantages and challenges faced by the multi-target intervention of natural products, this review is expected to provide a new perspective for in-depth understanding of the complex pathophysiology of PH, and offer theoretical basis and ideas for the development of new therapeutic strategies based on natural products, which are intended to reverse immune-metabolic imbalance and vascular remodeling. Ultimately, it will promote the translation of natural products from basic research to clinical practice, providing new hope for improving the prognosis of PH patients.

## 2 The immune-metabolic regulatory network in PH

### 2.1 Immune dysregulation

**FIGURE 1:**
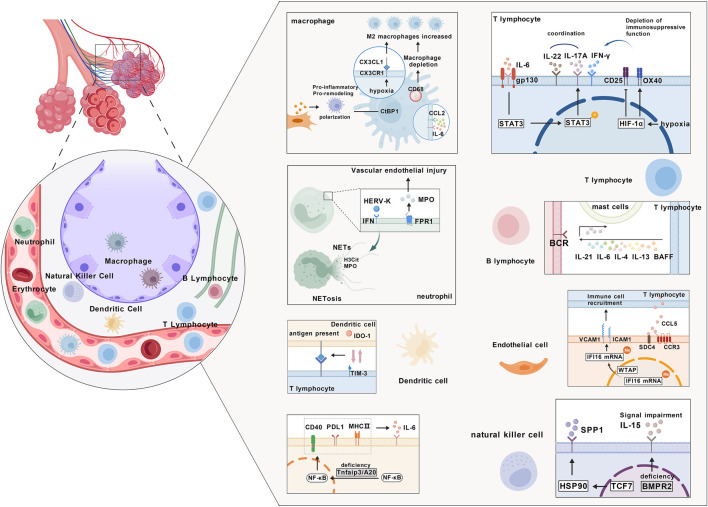
Immune system dysregulation in pulmonary hypertension: abnormal activation of innate immune cells, adaptive immune imbalance, and regulation of the inflammatory cytokine network; CX3CL1, C-X3-C Motif Chemokine Ligand 1; CX3CR1, C-X3-C Motif Chemokine Receptor 1; CD68, Cluster of Differentiation 68; CtBP1, C-Terminal Binding Protein 1; CCL2, C-C Motif Chemokine Ligand 2; gp130, Glycoprotein 130; CD25, Interleukin-2 Receptor Subunit Alpha; OX40, TNF Receptor Superfamily Member 4; STAT3, Signal Transducer and Activator of Transcription 3; HIF-1α, Hypoxia-Inducible Factor 1 Alpha; HERV-K, Human Endogenous Retrovirus K; IFN, Interferon; MPO, Myeloperoxidase; FPR1, Formyl Peptide Receptor 1; NETS, Neutrophil Extracellular Traps; BAFF, B Cell Activating Factor; IDO-1, Indoleamine 2,3-Dioxygenase 1; TIM-3, T-Cell Immunoglobulin and Mucin Domain-Containing Protein 3; VCAM1, Vascular Cell Adhesion Molecule 1; ICAM1, Intercellular Adhesion Molecule 1; CCL5, C-C Motif Chemokine Ligand 5; SDC4, Syndecan 4; CCR3, C-C Chemokine Receptor Type 3; CD40, Cluster of Differentiation 40; PDL1, Programmed Death-Ligand 1; MHC Ⅱ, Major Histocompatibility Complex Class II; SPP1, Secreted Phosphoprotein 1; HSP90, Heat Shock Protein 90; TCF7, Transcription Factor 7; BMPR2, Bone Morphogenetic Protein Receptor Type 2. The image was created with BioGDP.com (Jiang S et al., 2025).

The pathogenesis of PH is closely associated with immune system dysregulation. As summarized in Figure 1, this complex process involves the synergistic effects of abnormal activation of innate immune cells, imbalance of adaptive immunity, and disorder of inflammatory factor networks.

#### 2.1.1 Abnormal activation and polarization of innate immune cells

In the pathological process of PH, abnormal activation and polarization of innate immune cells are core links of immune dysregulation, whose mechanisms involve functional remodeling of various cell types and regulation of signaling pathways. As key participants, macrophages polarize toward pro-inflammatory/pro-remodeling phenotypes under the induction of metabolites and proteins secreted by adventitial fibroblasts, accompanied by mitochondrial metabolic reprogramming and activation of the CtBP1 pathway; inhibition of this pathway can reduce macrophage activation (Li M. et al., 2021). The polarization of M2 macrophages in PAH is unbalanced with that of M1 macrophages with CX3CR1 positive. The hypoxia microenvironment drives the increase of M2 macrophages, which is closely related to chemokine signaling pathways such as CX3CR1/cx3cl1, and then promotes vascular remodeling (Mao et al., 2022). In a mouse model with CD68-driven macrophage depletion, reduced macrophages lead to an imbalance in M1/M2 polarization, with a predominance of M2 type, inducing spontaneous PAH accompanied by PASMCs proliferation (Zawia et al., 2021). The impaired M2 polarization capacity of monocyte-derived macrophages from PAH patients after IL-4 stimulation suggests that macrophage polarization imbalance plays a key role in immune dysregulation in PAH (Zawia et al., 2021).

Abnormal activation of neutrophils and dendritic cells (DCs) also contributes to immune dysregulation in PH. Neutrophils activate the IFN antiviral signaling pathway through the endogenous retrovirus HERV-K envelope protein, enhancing the release of neutrophil extracellular traps (NETs) and the process of NETosis. Their extracellular vesicles carrying neutrophil elastase and HERV-K protein can induce PH in mice (Taylor et al., 2022). Deletion of the Tnfaip3/A20 gene in DCs activates the NF-κB pathway, leading to increased numbers of cDC1, cDC2, and monocyte-derived DCs (mo-DCs), as well as upregulated expression of surface activation markers MHC Ⅱ, CD40, and PDL1, thereby inducing PH in mice accompanied by pulmonary vascular remodeling, inflammatory cell infiltration, and release of cytokines such as IL-6. This pathological process depends on IL-6-mediated immune dysregulation (Koudstaal et al., 2020).

The stress response of natural killer (NK) cells represents a novel direction in the immune mechanisms of PH. In the lung tissues of PH patients and MCT-induced PH rat models, the proportion of stress-responsive NK cells is significantly increased. TCF7 is a key marker distinguishing high- and low-stress NK cells, with TCF7+ NK cells exhibiting stronger stress characteristics. TCF7 enhances the stress response of NK cells by regulating HSP90 within them, promoting the secretion of SPP1, which in turn promotes the proliferation and migration of PASMCs through paracrine effects, thereby participating in pulmonary vascular remodeling (Wu et al., 2025). Deficiency of BMPR2, a core mediator of NK cell homeostasis, can lead to NK cell dysfunction by impairing IL-15 signaling, which is involved in the pathogenesis of PH (Hilton et al., 2022). The abnormal activation of innate immune cells, as summarized in Figure 1, involves multi-dimensional mechanisms such as metabolic reprogramming, inflammasomes, complement pathways, and intercellular signal crosstalk, providing a rich theoretical basis for the development of immune-targeted therapeutic strategies for PH.

#### 2.1.2 Imbalance and autoreactivity of the adaptive immune system

Imbalance and autoreactivity of the adaptive immune system drive the progression of PH through multicellular interactions and molecular mechanisms. In patients with PAH, chemokines secreted locally by pulmonary vessels recruit B cells, which recognize self-antigens *via* B cell receptors and receive co-stimulatory signals from follicular helper T cells (Tfh) (IL-21), Th2 cells (IL-4/IL-6/IL-13), and mast cell cytokines. Meanwhile, B cell-activating factor (BAFF) enhances B cell survival signals, leading to the formation of perivascular tertiary lymphoid organs and germinal center reactions. Activated B cells differentiate into plasma cells, producing pathogenic autoantibodies (anti-endothelial cell antibodies, anti-AT1R/ETAR antibodies, anti-BMPR2 antibodies). These autoantibodies induce inflammation, vasoconstriction, and remodeling by directly binding to vascular cell targets, and activate the complement cascade and IgE-mast cell axis, amplifying pulmonary vascular inflammation and remodeling (Heukels et al., 2021; Jones et al., 2022; Sanges et al., 2024). Dysfunction of regulatory T cells (Tregs) is a key component of adaptive immune imbalance. Chronic hypoxia drives the conversion of Tregs to pro-inflammatory Th17 cells *via* the HIF-1α pathway, characterized by reduced CD25 expression, increased OX40 expression, and exhaustion of immunosuppressive function, which exacerbates Th1/Th17 cell polarization and the release of pro-inflammatory factors such as IL-17A and IFN-γ (Lantz et al., 2024). Abnormalities in antigen-presenting cells further amplify autoreactivity. Mo-DCs infiltrate the vascular wall and promote smooth muscle cell proliferation. In heritable PAH (HPAH), interactions between TIM-3^+^ T cells and IDO-1^+^ DCs are enhanced, activating effector T cells through the presentation of self-antigens (Ferrian et al., 2024). In idiopathic PAH patients, viral infections and adaptive immune dysregulation exhibit a synergistic effect. Increased Epstein-Barr virus load can activate CD4^+^ T cells *via* the PD-1/PD-L1 pathway, accompanied by an increase in Treg numbers but impaired function. Serum levels of pro-inflammatory factors such as IL-6 and IFN-γ are positively correlated with viral load (Tomaszewski et al., 2020; Tomaszewski et al., 2024). These mechanisms indicate that the autoreactivity of the adaptive immune system drives PH vascular inflammation and remodeling through multi-dimensional pathways, including B cell-T cell axis imbalance, Treg functional exhaustion, abnormal antigen presentation, and virus-immune interactions (Figure 1).

#### 2.1.3 Inflammatory factor network

The culmination of innate and adaptive immune dysregulation is the formation of a disordered inflammatory factor network, which acts as the final effector driving vascular pathology, as represented in the signaling cascade and cytokine interactions within Figure 1. In the process of immune dysregulation in PH, the inflammatory cytokine network exhibits complex regulatory mechanisms through cross-interactions of various cells and signaling pathways. At the level of innate immunity, neutrophils are recruited to lung tissues *via* formyl peptide receptor 1 (FPR1) and release inflammatory mediators such as myeloperoxidase (MPO), exacerbating vascular endothelial damage (Fadon-Padilla et al., 2024). The increased infiltration of M1-type macrophages, γδ T cells, and other cells activates the NLRP3 inflammasome by secreting pro-inflammatory factors like IL-1β and IL-6, promoting the maturation and release of IL-1β and IL-18, thereby driving pulmonary vascular remodeling (Xu et al., 2021; Mavrogiannis et al., 2022; Lian et al., 2023). In adaptive immunity, CD4^+^ T cells are activated through the IL-6/gp130/STAT3 signaling pathway, differentiate into Th17 cells, and secrete IL-17a. This, in synergy with IL-22, amplifies the inflammatory cascade. Among them, IL-22 participates in vascular remodeling by regulating the proliferation and fibrosis of vascular smooth muscle cells, and its high expression in patients’ serum can serve as a biomarker for disease progression (Chen J. Y. et al., 2020; Ishibashi et al., 2024; Klein et al., 2024). In epigenetic regulation, WTAP-mediated m^6^A methylation stabilizes IFI16 mRNA, further amplifying inflammatory signals, promoting the expression of endothelial cell adhesion molecules VCAM1 and ICAM1, and enhancing immune cell recruitment (Rao et al., 2025). In addition, the chemokine CCL5, as a key effector molecule of T cells and NK cells, mediates interactions between immune cells, vascular endothelial cells, and smooth muscle cells by binding to receptors such as CCR3 and SDC4, exacerbating vascular occlusion (Fadon-Padilla et al., 2024; Li et al., 2024c). Inflammatory factors such as IL-6, IL-22, and CCL5 are not only effector molecules of immune activation but also regulators of metabolic disorders. The imbalance of their dynamic equilibrium forms a vicious cycle with pulmonary vascular remodeling and right heart dysfunction through signaling pathways such as TGF-β and JAK-STAT, providing multi-dimensional intervention targets for PH-targeted therapy.

### 2.2 Metabolic remodeling

**FIGURE 2:**
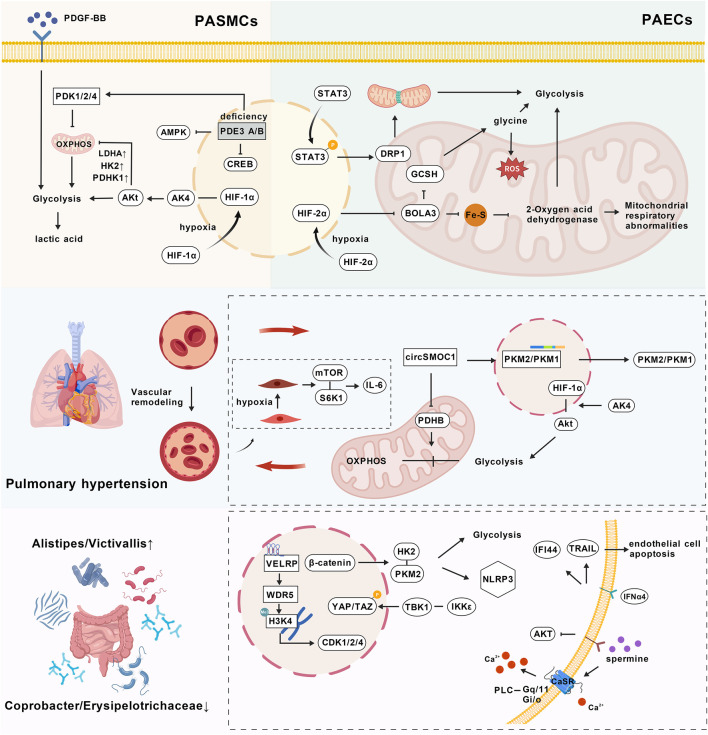
Metabolic remodeling in pulmonary hypertension: cellular metabolic reprogramming, metabolites, signaling pathways, and vascular remodeling; PASMCs, Pulmonary Arterial Smooth Muscle Cells; PAECS, Pulmonary Arterial Endothelial Cells; PDGF-BB, Platelet-Derived Growth Factor BB; PDK1/2/4, Pyruvate Dehydrogenase Kinase 1/2/4; OXPHOS, Oxidative Phosphorylation; LDHA, Lactate Dehydrogenase A; HK2, Hexokinase 2; PDHK, Pyruvate Dehydrogenase Kinase; Akt, Protein Kinase B; AK4, Adenylate Kinase 4; AMPK, AMP-Activated Protein Kinase; PDE3 A/B, Phosphodiesterase 3 A/B; CREB, cAMP Response Element-Binding Protein; HIF-1α, Hypoxia-Inducible Factor 1 Alpha; STAT3, Signal Transducer and Activator of Transcription 3; HIF-2α, Hypoxia-Inducible Factor 2 Alpha; DRP1, Dynamin-Related Protein 1; GCSH, Glycine Cleavage System Protein H; ROS, Reactive Oxygen Species; mTOR, Mammalian Target of Rapamycin; S6K1, Ribosomal Protein S6 Kinase 1; circsMoC1, Circular RNA MoC1; PDHB, Pyruvate Dehydrogenase E1 Beta Subunit; PKM2/PKM1, Pyruvate Kinase M2/Pyruvate Kinase M1; VELRP, VEGF-Regulated Protein; WDR5, WD Repeat Domain 5; H3K4, Histone H3 Lysine 4; CDK1/2/4, Cyclin-Dependent Kinase 1/2/4; YAP/TAZ, Yes-Associated Protein/Transcriptional Co-Activator with PDZ-Binding Motif; HK2, Hexokinase 2; PKM2, Pyruvate Kinase M2; NLRP3, NOD - like receptor family pyrin domain containing 3; TBK1, TANK-Binding Kinase 1; IKKε, IκB Kinase Epsilon; IFI44, Interferon-Inducible Protein 44; TRAIL, Tumor Necrosis Factor-Related Apoptosis-Inducing Ligand; IFNα4, Interferon Alpha 4; CaSR, Calcium-Sensing Receptor; Gq/11-PLC, Gq/11 G-Protein-Phospholipase C; The image was created with BioGDP.com (Jiang S. et al., 2025).

The occurrence and progression of PH are closely associated with multi-cellular metabolic reprogramming, which collectively drives the pathological process of the disease through abnormal switching of cellular energy metabolic pathways, regulation of key metabolites and signaling pathways, and bidirectional interactions between metabolic disorders and vascular remodeling (Figure 2).

#### 2.2.1 Cellular mechanisms of metabolic reprogramming

The core pathological feature of PH is metabolic reprogramming in cells such as PASMCs and PAECs. Figure 2 highlights the cell-autonomous metabolic mechanisms within PASMCs and PAECs that initiate this reprogramming. This metabolic reprogramming is mainly manifested as abnormal shifts in cellular energy metabolism pathways, including enhanced glycolysis and mitochondrial dysfunction, which collectively drive the pathological process of pulmonary artery vascular remodeling.

PASMCs are the core cells involved in metabolic reprogramming. The phenotypic transformation of PDGF-BB-induced PASMCs is accompanied by abnormalities in pyruvate metabolism and glycolysis/gluconeogenesis pathways, with changes in the levels of metabolites such as pyruvate and lactic acid providing metabolic support for cell proliferation (Zhang M.-J. et al., 2024). Deletion of Pde3a and Pde3b in PASMCs can inhibit mitochondrial oxidative phosphorylation and enhance glycolysis by downregulating the AMPK/CREB signal and upregulating PDK1/2/4 (Krause et al., 2024).

The metabolic reprogramming of PASMCs is mainly driven by chronic hypoxia, which upregulates adenylate kinase 4 (AK4) in a HIF-1α-dependent manner. AK4 forms a positive feedback loop with HIF-1α and activates the Akt signaling pathway, promoting the metabolic shift from mitochondrial oxidative phosphorylation to glycolysis. This is accompanied by increased expression of glycolytic enzymes such as HK2, LDHA, and PDHK1, accumulation of lactic acid, and enhanced proliferative capacity of PASMCs, thereby participating in pulmonary vascular remodeling (Wujak et al., 2021).

The mechanism of metabolic reprogramming in PAECs is closely related to the downregulation of BOLA3. The expression of BOLA3 is reduced due to HIF-2α-dependent transcriptional inhibition, and its deletion disrupts the integrity of iron-sulfur (Fe-S) clusters, affects the function of two-oxoacid dehydrogenases containing lipoic acid, and regulates glycolysis and mitochondrial respiration. Meanwhile, BOLA3 deficiency can downregulate glycine cleavage system protein H (GCSH), leading to intracellular glycine accumulation, inducing a metabolic shift towards glycolysis and fatty acid oxidation, generating reactive oxygen species, promoting endothelial cell proliferation, survival, and vasoconstriction, and reducing angiogenic capacity (Yu et al., 2019). STAT3 phosphorylation promotes glycolytic metabolic remodeling and apoptosis resistance in pulmonary artery endothelial cells by regulating DRP1-mediated mitochondrial fission, forming a regulatory axis involving signaling pathways, mitochondrial dynamics, and metabolic reprogramming (Zhang H. et al., 2024).

Adventitial fibroblasts regulate the phenotypic transformation of smooth muscle cells by secreting laminin, hepatocyte growth factor, *etc.*, accompanied by reduced mitochondrial content, membrane potential hyperpolarization, and enhanced glycolysis, revealing that intercellular interactions induce metabolic reprogramming by remodeling the metabolic microenvironment (Crnkovic et al., 2025).

#### 2.2.2 Key metabolites and signaling pathways

A variety of key metabolites and signaling pathways play important regulatory roles in the process of metabolic remodeling in PH. These cellular metabolic shifts alter the metabolic landscape, leading to the dysregulation of key metabolites and signaling pathways, as depicted in Figure 2. Gut microbiota metabolism is closely related to the risk of PH. The genera *Alistipes* and *Victivallis* can increase the risk of the disease, while *Coprobacter* and Erysipelotrichaceae have protective effects. Intestinal IFNα4 drives the progression of PAH by inducing the expression of IFI44 in CD8^+^ T cells and promoting TRAIL-mediated endothelial cell apoptosis, revealing the key role of metabolic signals in the gut-immune axis (Li et al., 2024d; Ruffenach et al., 2024).

In terms of energy metabolism reprogramming, the deletion of Pde3a and Pde3b inhibits mitochondrial oxidative phosphorylation and promotes glycolysis in PASMCs by downregulating the AMPK/CREB signal and upregulating PDK1/2/4. In contrast, β-catenin activates key glycolytic enzymes such as HK2 and PKM2 and promotes the activation of the NLRP3 inflammasome in macrophages. These processes regulate metabolism and inflammation through the PDE3-AMPK-glycolysis pathway and the Wnt/β-catenin-glycolysis axis, respectively (Krause et al., 2024; Meng et al., 2024). Spermine can promote the proliferation of PASMCs by affecting the inactivation of the AKT pathway. It can also activate the calcium-sensing receptor (CaSR) and trigger downstream signaling pathways such as Gq/11-PLC and Gi/o, leading to an increase in intracellular calcium concentration and vascular remodeling (Ding et al., 2022; Zhang J. et al., 2024).

Epigenetic regulatory pathways play an important role in metabolic remodeling. Non canonical IKB kinases TBK1/IKK ε promotes PASMCs proliferation and vascular remodeling by regulating YAP/TAZ signaling pathway (Aravamudhan et al., 2024). The long noncoding RNA VELRP enhances H3K4 trimethylation and upregulates CDK1/2/4 by binding WDR5, which drives metabolic abnormalities through TBK1/IKKε-YAP/TAZ axis and VELRP/WDR5/CDK signaling, respectively (Aravamudhan et al., 2024; Liu et al., 2024). Metabolic remodeling in PH involves multi-dimensional signaling networks such as gut microbiota-immune metabolism, epigenetics, energy metabolism, and ion channels. Targeting key metabolites such as SCFAs and spermine, as well as pathways such as AKT, Wnt/β-catenin, and CaSR, provides important directions for disease treatment.

#### 2.2.3 Metabolic disorders and vascular remodeling

Metabolic disorders and vascular remodeling exhibit a bidirectional interaction in PH, which forms the core of Figure 2. Metabolic abnormalities directly drive vascular remodeling through energy metabolic reprogramming, substrate competition, and signaling pathway activation, while the process of vascular remodeling further exacerbates metabolic imbalance, forming a pathological cascade reaction.

circSMOC1 drives the uncoupling of glycolysis and mitochondrial oxidative phosphorylation in PASMCs by regulating PKM2/PKM1 splicing and PDHB expression, thereby promoting proliferation and migration (Lu et al., 2022). AK4 activates glycolytic metabolism *via* the Akt/HIF-1α pathway and inhibits mitochondrial function, forming a Warburg effect (Wujak et al., 2021). PDGF-BB-induced phenotypic transformation is accompanied by pyruvate metabolic disorder, and the accumulation of metabolites provides material basis for cell proliferation (Zhang M.-J. et al., 2024). There are abnormalities in fatty acid oxidation, tricarboxylic acid cycle and nucleotide metabolism in the right ventricle of patients with obesity related PAH. The metabolites such as orotic acid, bile acid and acylcarnitine are closely related to PH, revealing the role of metabolic dysfunction in vascular remodeling (Knauss et al., 2025). In addition, the high uptake of proline and glucose by vascular endothelium and medial biomass can promote extracellular matrix synthesis and vascular wall thickening (Wertheim et al., 2023).

Endoplasmic reticulum stress, calcium disorder, and mitochondrial dysfunction synergistically drive the phenotypic transformation of vascular cells. Cells with imbalanced proliferation and apoptosis maintain a proliferative advantage through metabolic reprogramming, characterized by enhanced glycolysis and disrupted redox homeostasis (Krause et al., 2024; Zhou et al., 2024). Hypoxia-induced senescence of PASMCs releases IL-6 *via* the mTOR/S6K1 pathway, and paracrine signals activate the metabolic-inflammatory network in adjacent cells, forming a metabolism-driven effect of senescence-associated secretory phenotype (Wang et al., 2021). This bidirectional mechanism provides a multi-level theoretical basis for targeted metabolic intervention.

### 2.3 Immune-metabolic interactive regulatory network

**FIGURE 3:**
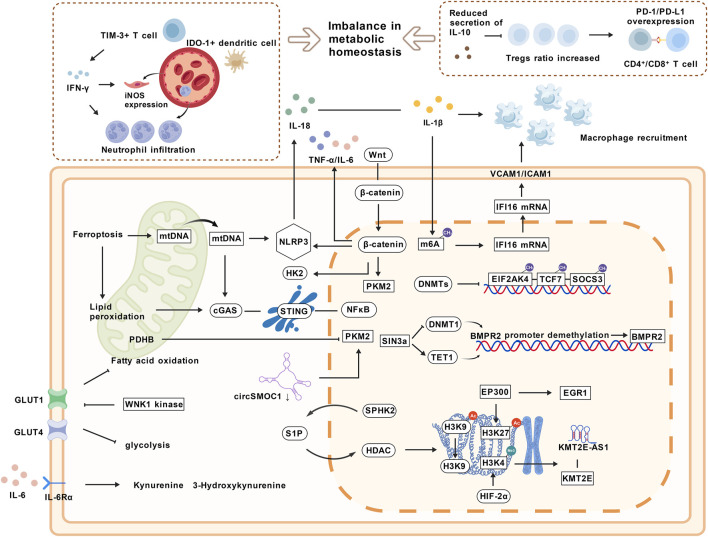
The Immuno-Metabolic Crosstalk Regulatory Network in Pulmonary Hypertension. TIM-3^+^ T cell, T-cell Immunoglobulin and Mucin-domain containing-3. IFN-γ, Interferon-gamma; iNOS, Inducible Nitric Oxide Synthase; IDO-1, Indoleamine 2,3-Dioxygenase 1; PD-1/PD-L1, Programmed Death 1/Programmed Death Ligand 1; VCAM1/ICAM1, Vascular Cell Adhesion Molecule 1/Intercellular Adhesion Molecule 1; mtDNA, mitochondrial DNA; NLRP3, NOD - like receptor family pyrin domain containing 3; HK2, Hexokinase 2; cGAS, Cyclic GMP-AMP Synthase; STING, Stimulator of Interferon Genes; PKM2, Pyruvate Kinase M2; NFκB, Nuclear Factor Kappa-light-chain-enhancer of Activated B Cells; DNMTs, DNA Methyltransferases; EIF2AK4, Eukaryotic Initiation Factor 2 Alpha Kinase 4; TCF7, Transcription Factor 7; SOCS3, Suppressor of Cytokine Signaling 3; PDHB, Pyruvate Dehydrogenase Beta Subunit; TET1, Ten-Eleven Translocation 1; BMPR2, Bone Morphogenetic Protein Receptor Type 2; GLUT, Glucose Transporter; WNK1, With-No-Lysine Kinase 1; S1P, Sphingosine-1-Phosphate; SPHK2, Sphingosine-1-Phosphate; HDAC, Histone Deacetylase; EP300, E1A Binding Protein P300; EGR1, Early Growth Response 1; KMT2E, Lysine Methyltransferase 2E; HIF-2α, Hypoxia-Inducible Factor 2 Alpha. The image was created with BioGDP.com (Jiang S. et al., 2025).

In the pathological process of PH, the interactive regulatory network between immunity and metabolism collectively drives vascular remodeling and the formation of an inflammatory microenvironment through multi-dimensional molecular mechanisms. As outlined in Figure 3, this network operates through several key pathways: the regulation of immune cells by metabolites, the remodeling of metabolic pathways by immune signals, the activation of the mitochondria-inflammasome axis, and the cascade effects of epigenetic regulation.

#### 2.3.1 Regulation of immune cells by metabolites

Metabolites participate in vascular remodeling and right ventricular dysfunction in PH by regulating energy metabolism and signaling pathways of immune cells. Disorders of glucose and fatty acid metabolism are core links in the remodeling of the immune microenvironment in PH. The With No Lysine Kinase 1 (WNK1) kinase inhibitor WNK463 regulates right ventricular glucose metabolism by inhibiting GLUT1/4 expression and protein glycosylation, while affecting mitochondrial fatty acid oxidation pathways, thereby improving metabolic disorders and alleviating right ventricular dysfunction. This suggests that metabolites such as glucose and acylcarnitines may regulate the inflammatory microenvironment by activating immune-related signals such as AMPK (Prisco et al., 2021). Tryptophan metabolites change significantly in PAH. The levels of metabolites such as kynurenine and 3-hydroxykynurenine produced after activation of the kynurenine pathway are elevated, which is associated with disease severity. The IL-6/IL-6Rα signal promotes the formation of an immune-inflammatory microenvironment by inducing KP activation in pulmonary vascular cells (Cai et al., 2022). In PAH patients, reduced monocyte infiltration is accompanied by abnormalities in the ICAM-1/ITGAL-ITGB2 pathway. Amino acids such as threonine and alanine, lipids, and glycolytic intermediates (e.g., lactic acid) participate in the progression of PH by regulating energy metabolism in immune cells (Xu et al., 2022; Zhong et al., 2023).

Dysbiosis of the intestinal flora exacerbates immune dysregulation in PH through an imbalance of metabolites. Intestinal dysbiosis is characterized by a reduction in the flora producing anti-inflammatory metabolites (such as short-chain fatty acids and secondary bile acids) and their encoding genes, along with an enrichment of flora producing the pro-inflammatory metabolite trimethylamine, resulting in metabolic imbalance. This imbalance exacerbates pulmonary vascular immune dysregulation and remodeling by weakening the regulatory T cell-promoting and inflammation-inhibiting effects of short-chain fatty acids, reducing the inhibitory effect of secondary bile acids on pro-inflammatory cytokines, and enhancing the activation of macrophages by trimethylamine-related metabolites (Moutsoglou et al., 2023). Similarly, in a rat model of PH induced by chronic hypoxia, intestinal dysbiosis leads to a decrease in the short-chain fatty acids valeric acid and isovaleric acid, as well as the accumulation of pro-inflammatory metabolites. This exacerbates immune dysregulation by affecting macrophage polarization and the balance of T cell subsets, thereby participating in the pathological process of PH (Cao et al., 2024).

Metabolites achieve fine regulation of immune cell functions and inflammatory signals through multiple pathways such as metabolic reprogramming, tryptophan metabolism, and intestinal flora metabolism regulation.

#### 2.3.2 Remodeling of metabolic pathways by immune signals

Abnormal activation of immune signaling pathways affects the progression of PH by remodeling metabolic pathways. Activation of the NLRP3 inflammasome directly exacerbates inflammatory responses and vascular remodeling through caspase-1-dependent maturation of IL-1β/IL-18 and macrophage recruitment (Mavrogiannis et al., 2022). Meanwhile, IL-1β upregulates IFI16 expression *via* m6A epigenetic modification, inducing inflammatory apoptosis of PAECs, revealing the epigenetic regulation of metabolic pathways by inflammatory factors (Rao et al., 2025). The Wnt/β-catenin pathway bidirectionally regulates immunometabolism: on one hand, it promotes glycolysis in macrophages and activation of NLRP3; on the other hand, it upregulates key glycolytic enzymes (HK2, PKM2) and inflammatory factors (TNF-α, IL-6), driving the proliferation of PASMCs (Meng et al., 2024).

Immune cell subsets are closely related to the regulation of the metabolic microenvironment. In idiopathic PAH, genes such as IGF1 and KARS participate in metabolic remodeling by regulating glycolysis in fibroblasts (Gao et al., 2025). In HPAH, TIM-3^+^ T cells and IDO-1^+^ dendritic cells are enriched around blood vessels, and they promote neutrophil infiltration and endothelial cell iNOS expression by secreting IFN-γ, exacerbating metabolic disorders and vascular remodeling (Ferrian et al., 2024).

Immune checkpoint molecules participate in PH progression through interactive regulation of immune cell functions and metabolic pathways. In patients with idiopathic PAH, the expression of PD-1/PD-L1 on the surface of peripheral blood CD4^+^ and CD8^+^ lymphocytes are significantly upregulated, the proportion of Tregs is increased, and IL-10 secretion is reduced, suggesting that excessive activation of the PD-1/PD-L1 axis synergizes with Treg dysfunction to exacerbate immune dysregulation and affect metabolic homeostasis (Tomaszewski et al., 2024). Abnormal expression of PDCD1 and CD274 in T cell subsets of PAH patients is associated with enrichment of T cell activation and cytotoxic pathways (Jiang P. et al., 2025). In addition, immune checkpoint molecules regulate metabolic crosstalk between immune cells and vascular smooth muscle cells through intercellular communication networks such as MIF-CD44 (Jiang P. et al., 2025). The mechanisms by which immune signals remodel metabolic pathways through multiple dimensions including inflammatory factors, signal transduction, and intercellular communication emphasize the core role of immune-metabolic crosstalk in the pathogenesis of PH.

#### 2.3.3 Mitochondria-inflammasome axis in the immune-metabolic interactive regulatory network

In PH, the mitochondria-inflammasome axis drives the pathological process through multiple mechanisms. Mitochondrial metabolic abnormalities are manifested as downregulation of the electron transport chain and dysregulation of the citric acid cycle homeostasis. 3-phenyllactic acid, as a potential biomarker associated with mitochondrial dysfunction, its metabolites can promote PAH progression through the inflammatory metabolic network (Zhang X. et al., 2025). Abnormalities in mitochondrial dynamic regulation are significant in PASMCs of patients with PAH. Prostacyclin analogs reverse excessive mitochondrial fission and cell proliferation phenotypes by inhibiting phosphorylation of the mitochondrial fission protein DRP1, revealing the impact of dynamic balance on immune-metabolic crosstalk (Abu-Hanna et al., 2023).

mtDNA released from mitochondrial damage acts as a danger signal, which can activate the NLRP3 inflammasome, promote caspase-1-mediated cleavage of IL-1β/IL-18 and macrophage recruitment. Inhibition of this pathway can reduce vascular remodeling and inflammatory responses (Mavrogiannis et al., 2022). In addition, ferroptosis induces lipid peroxidation and mtDNA release in pulmonary artery endothelial cells, activates the cGAS-STING-NFκB pathway, drives the inflammatory phenotype of monocytes and proliferation of PASMCs, while inhibition of ferroptosis can alleviate related pathologies (Kazmirczak et al., 2024). The neuropeptide CGRP protects mitochondrial integrity through the PKA pathway, inhibits mtDNA release and activation of the cGAS-STING-NFκB pathway, and reduces PDGF-BB-induced inflammatory factor secretion and cell proliferation, further highlighting the core role of the mitochondria-inflammasome axis (Yan et al., 2024). The interaction between mitochondrial dysfunction and inflammatory signals regulates the immune-metabolic network through multiple pathways, becoming a core driver of PH vascular remodeling.

#### 2.3.4 Epigenetic regulation in the immune-metabolic interactive network

In the immune-metabolic interactive regulatory network of PH, epigenetic mechanisms serve as key hubs connecting metabolic abnormalities and immune imbalance through DNA methylation, histone modification, and noncoding RNA regulation.

DNA methylation affects the expression of immune-metabolic related genes by regulating the methylation status of gene promoters in PH. In PAH, DNA methylation mediates CpG site methylation in gene promoters and enhancer regions *via* DNA methyltransferases (DNMTs), leading to hypermethylation or hypomethylation of immune-metabolic related genes such as EIF2AK4, TCF7, and SOCS3 with concomitant abnormal expression, which enriches functions like T cell differentiation and participates in the pathological process of PAH (Benincasa et al., 2023; Hindmarch et al., 2025). Hypermethylation of the BMPR2 promoter is associated with its downregulated expression. The transcription factor SIN3a enhances the activity of the DNA demethylase TET1 by inhibiting the DNA methyltransferase DNMT1 and histone methyltransferase EZH2, thereby reducing the methylation level of the BMPR2 promoter, restoring its expression, and inhibiting the proliferation of PASMCs (Bisserier et al., 2021).

Histone modification plays a crucial role in epigenetic regulation. Abnormalities in histone deacetylases (HDACs) can drive immune dysregulation in PAH by downregulating Foxp3^+^ Tregs. The HDAC inhibitor SAHA can improve PAH by restoring histone acetylation, inducing Treg transformation, and regulating immune-vascular interaction-related genes such as PD-1 and BMPR-2 (Chen et al., 2023). Sphingosine kinase 2 (SPHK2) can activate HDAC through the production of S1P, leading to histone H3K9 deacetylation and epigenetic remodeling of PASMCs, thereby promoting PAH vascular remodeling. Inhibition of SPHK2 can reverse abnormal proliferation of PASMCs by restoring histone acetylation (Ranasinghe et al., 2023). Additionally, early growth response factor 1 (EGR1) can bind to the promoter region of histone acetyltransferase EP300, upregulate EP300 expression, and enhance histone H3K27 acetylation, thereby inhibiting inflammation and apoptosis of PAECs and alleviating hypoxia-induced PAH (Yang et al., 2023).

Long noncoding RNAs (lncRNAs) and circular RNAs (circRNAs) regulate metabolic and immune signals through epigenetic and post-transcriptional regulatory mechanisms. The lncRNA KMT2E-AS1 can bind to the histone methyltransferase KMT2E, enhance histone H3K4 trimethylation, and drive metabolic reprogramming *via* HIF-2α. Its single nucleotide variation (SNV) rs73184087 (G allele) promotes PAH progression through chromatin interaction (Tai et al., 2024). The circRNA circSMOC1 is significantly downregulated in the pulmonary arteries of PAH rats. It inhibits PKM precursor splicing and upregulates PKM2 in the nucleus, while adsorbing miR-329-3p in the cytoplasm to regulate PDHB expression, mediating smooth muscle cell metabolic remodeling and vascular remodeling through a dual mechanism (Lu et al., 2022). Epigenetic mechanisms dynamically regulate gene expression through multiple dimensions, integrating immune and metabolic signals.

#### 2.3.5 Cell-specific mechanisms of immune-metabolic crosstalk

In PH, the crosstalk between immunity and metabolism exhibits a high degree of cell specificity. As summarized in Table 1, macrophages, T lymphocytes, and vascular wall cells, through their distinct bidirectional immunometabolic regulatory networks, predominantly drive the formation of the inflammatory microenvironment, immune imbalance, and vascular remodeling, thereby promoting the disease’s pathological progression.

**TABLE 1:** Immune-metabolic coupling mechanisms of key cell types in pulmonary hypertension.

Cell type	Core metabolic alterations	Immunological consequences	PH pathology
Macrophage	1. M1 Polarization-Associated: Enhanced glycolysis (HK2, PKM2, Lactate ↑); Activated glutamine metabolism (GLS1 expression ↑) 2. M2 Polarization-Associated: Enhanced pentose phosphate pathway (PPP) flux (G6PD activity ↑) 3. Microenvironment-Driven: sEVs containing complement C3 drive glycolysis	1. M1 Polarization-Associated: Promotes pro-inflammatory cytokine expression, exacerbating inflammation; Activates the NLRP3 inflammasome. 2. M2 Function Support: PPP may support specific functions of M2 macrophages.	1. Drives vascular inflammation. 2. Exacerbates PASMC proliferation and migration. 3. Promotes vascular remodeling.
T Cell (Th17/Treg)	1. Tryptophan Metabolic Pathway Skewing: Activated kynurenine pathway, elevated plasma kynurenine levels. 2. Aberrant Epigenetic Regulation: HDAC activity affects Treg function.	1. Disrupted Immune Balance: Leads to Treg/Th17 functional and proportional imbalance; Treg functional deficiency (expansion of non-suppressive subtypes). 2. Loss of Immunosuppressive Function: Overall immunosuppressive function is compromised.	1. Directly drives immune imbalance and disease progression. 2. High kynurenine concentration predicts adverse clinical outcomes.
PASMC	1. Energy Metabolic Reprogramming: Significantly enhanced glycolysis. 2. Mitochondrial Dysfunction: mtDNA release.	Shapes Immune Microenvironment: Lactate release activates the Akt pathway, promoting remodeling; mtDNA release activates the cGAS-STING pathway, triggering inflammation and immune cell infiltration.	1. Drives proliferative vascular obliteration. 2. Actively recruits and activates immune cells, forming a positive feedback loop.
PAEC	Metabolic Abnormalities: Mitochondrial dysfunction, increased ROS production; Potentially enhanced glycolysis.	Initiates Immune Response: Releases metabolites such as mtDNA and lactate; Promotes endothelial inflammation and macrophage polarization.	1. Causes endothelial dysfunction. 2. Initiates and amplifies inflammatory responses.

A bidirectional regulation exists between the polarization state (M1/M2) of macrophages and their metabolic programs, collectively contributing to inflammation and vascular remodeling in PH. Enhanced glycolysis is a key factor driving macrophages toward the pro-inflammatory M1 phenotype. Activation of the β-catenin signaling pathway upregulates HK2, PKM2, and lactate levels, thereby promoting inflammatory cytokine expression and exacerbating the proliferation and migration of PASMCs (Meng et al., 2024). In the MCT-induced PAH model, increased expression of glutaminase 1 (GLS1) is observed; inhibiting GLS1 reduces M1 polarization and NLRP3 inflammasome activation, ameliorating vascular remodeling (Chen X. et al., 2025). Concurrently, the activity of G6PD, a key enzyme in the pentose phosphate pathway (PPP), is enhanced in hypoxic PH. Its inhibition reduces M2 macrophage markers, suggesting that PPP metabolic flux may support specific functions of M2 macrophages (Hashimoto and Gupte, 2022). Macrophage polarization is also modulated by the microenvironment. PH fibroblasts drive pro-inflammatory phenotypes and glycolysis in macrophages *via* small extracellular vesicles (sEVs) containing complement C3 (Kumar et al., 2021).

Different T cell subsets, particularly Th17 cells and regulatory Tregs, exhibit significant metabolic and functional imbalances that directly drive PH progression. Activation of the kynurenine pathway of tryptophan metabolism is directly linked to the Treg/Th17 imbalance. Elevated plasma kynurenine levels in PAH patients correlate positively with the iTreg/Th17 ratio, and high kynurenine concentrations predict adverse clinical outcomes (Jasiewicz et al., 2016). Notably, Tregs in PH not only show numerical imbalance but also altered function and subtype composition, with expansion of non-suppressive Treg subtypes leading to an overall defect in immunosuppressive function (Sada et al., 2016). Histone deacetylase (HDAC)-dependent epigenetic modifications can restore the function of Foxp3+ Tregs. The HDAC inhibitor SAHA can induce the conversion of T cells into functional Tregs and alleviate pulmonary hypertension, confirming the central role of metabolic-epigenetic crosstalk in regulating T cell function (Chen et al., 2023).

**FIGURE 4:**
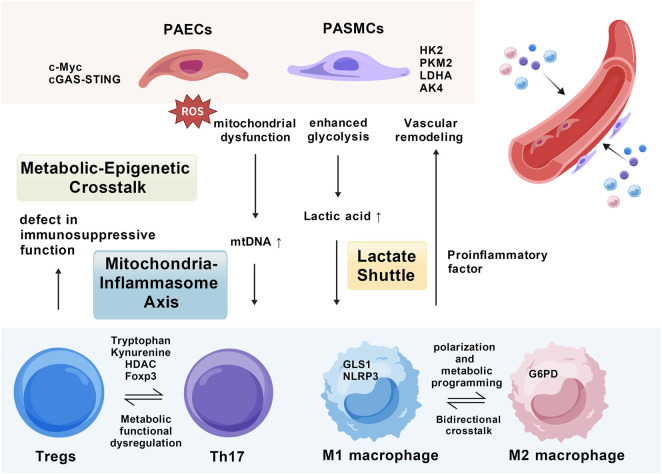
Schematic diagram of the multicellular immunometabolic dialogue network in pulmonary hypertension. PASMCs, pulmonary arterial smooth muscle cells. PAECs, pulmonary arterial endothelial cells; mtDNA, mitochondrial DNA; ROS, reactive oxygen species; HK2, hexokinase 2; PKM2, pyruvate kinase M2; LDHA, lactate dehydrogenase A; AK4, adenylate kinase 4; GLS1, glutaminase 1; G6PD, glucose-6-phosphate dehydrogenase; cGAS-STING, cyclic GMP-AMP synthase-stimulator of interferon genes; HDAC, histone deacetylase; Foxp3, forkhead box P3; The image was created with BioGDP.com (Jiang S. et al., 2025).

In PH, the metabolic reprogramming of PASMCs and PAECs actively shapes the immune microenvironment through the release of specific metabolites. PASMCs exhibit enhanced glycolysis and mitochondrial dysfunction, leading to increased lactate production and mtDNA release. Lactate promotes vascular remodeling by activating the Akt signaling pathway, while mtDNA activates the cGAS-STING pathway, triggering inflammatory responses and immune cell infiltration (Li J. et al., 2024; Wu et al., 2024). Similarly, metabolic abnormalities in PAECs, such as mitochondrial dysfunction and increased ROS production, promote endothelial inflammation and macrophage polarization through the release of metabolites like mtDNA and lactate (Geng et al., 2025; Rao et al., 2025). As illustrated in Figure 4, these metabolic-immune interactions further regulate the balance of T cell subsets (Th17/Treg) and macrophage polarization, forming a positive feedback loop that exacerbates PH progression (Wei et al., 2022; Han et al., 2025).

## 3 Natural products regulating PH

### 3.1 Immunomodulatory natural products

The immunomodulatory natural products summarized in Supplementary Table 1, including alkaloids, flavonoids, and compound formulations, demonstrate therapeutic potential across various PH models by targeting specific immune pathways.

#### 3.1.1 Targeting innate immune cells

Several natural products exert their therapeutic effects in PH by targeting the activation and function of innate immune cells.

Halofuginone is a quinazoline alkaloid extracted from *Hydrangea febrifuga*. It inhibits pulmonary tissue inflammatory responses by suppressing the TGF-β1/Smad signaling pathway. In a rat model of PH induced by hypoxia at 6,000 m altitude, halofuginone blocks the inflammatory cascade by inhibiting TGF-β1 expression and Smad2/3 phosphorylation, significantly reducing the levels of IL-1β, IL-6, and TNF-α in lung tissues, inhibiting the infiltration of neutrophils and lymphocytes, improving inflammatory damage to vascular walls, and decreasing the right ventricular hypertrophy index and pulmonary vascular wall thickness (Wang et al., 2025).

Notopterol is one of the main active components extracted from *Hansenia weberbaueriana* (a plant of the Apiaceae family) and belongs to phthalide compounds. Notopterol can significantly reduce the protein expression of pro-inflammatory factors IL-1β and IL-6 in the lung tissues of MCT-induced PAH rats and decrease the number of inflammatory macrophages, and its anti-inflammatory effect may be related to the inhibition of NF-κB-mediated inflammatory signals (Huang et al., 2022).

Fermented cordyceps powder is mycelium extracted from Cordyceps sinensis and has been proven to have similar active components and pharmacological effects to natural Cordyceps sinensis. It downregulates the expression of inflammatory factors such as IL-1β and IL-6 by inhibiting the p38 MAPK/NF-κB signaling pathway, thereby reducing pulmonary vascular inflammatory responses and abnormal proliferation of PASMCs (Li et al., 2023).

#### 3.1.2 Regulating adaptive immunity

Beyond innate immunity, certain natural products ameliorate PH by directly modulating the adaptive immune response, particularly T cell function and infiltration.

Ginsenoside Rh1 is an active component extracted from *Panax ginseng* C. A. Mey. (a plant of the Araliaceae family). Ginsenoside Rh1 interacts with six basement membrane-related genes such as ITGAV and ITGA4, which are closely related to the activity and recruitment of immune cells such as Tregs, Th1 cells, mast cells, and macrophages. Ginsenoside Rh1 may target these basement membrane-related genes to regulate the infiltration and function of immune cells, thereby exerting a role in improving PAH (Li and Zhang, 2023).

Perillyl alcohol is a limonene monoterpenoid compound. When used in combination with quercetin, perillyl alcohol can upregulate miR-204, inhibit the Src–STAT3–NFAT pathway, reduce inflammatory cell infiltration, regulate the balance between apoptosis and cell proliferation, and exert anti-oxidative stress effects, thereby improving PH (Rajabi et al., 2020; Kordestani et al., 2024).

#### 3.1.3 Inhibiting inflammatory factor networks

Tetramethylpyrazine is an alkaloid extracted from *Conioselinum anthriscoides* Chuanxiong. It reduces the release of inflammatory factors in lung tissues by inhibiting the HMGB1/endoplasmic reticulum (ER) stress pathway. In MCT-induced PAH rats, tetramethylpyrazine significantly decreases the levels of inflammatory factors such as IL-1β and IL-6 in lung tissues and alleviates inflammatory cell infiltration in vascular walls. Its mechanism is related to inhibiting the PERK/ATF4 signaling axis activated by HMGB1, reducing downstream SIAH2 expression, and restoring HIPK2 levels (Zhang Q. et al., 2023).

Isorhamnetin and baicalin are both flavonoids, which are the main active components of *Hippophae rhamnoides* L. and *Scutellaria baicalensis* Georgi, respectively. Both inhibit the expression of inflammatory factors such as TNF-α and IL-6 by activating the BMPR2/Smad signaling pathway (Chang et al., 2020; Xue et al., 2021). Baicalin can also block the TNF-α/NF-κB pathway, collectively regulating immune-inflammatory responses and inhibiting the proliferation of PASMCs (Xue et al., 2021).

#### 3.1.4 Compound formulations with multi-target immunoregulation

Compound natural products exhibit unique advantages in regulating immune-inflammatory pathways through the synergistic effects of multiple components and multiple targets. Buyang Huanwu Decoction inhibits abnormal proliferation and migration of vascular smooth muscle cells and reverses pulmonary vascular remodeling by activating the PI3K-Akt-eNOS pathway and restoring NO signaling. Meanwhile, it targets the TNF signaling pathway to alleviate pulmonary vascular inflammation (Chen Y. et al., 2021). The active components in Xinmai’an extract, such as ginsenoside Rg1, paeoniflorin, and tanshinone IIA, collectively block MAPK signal transduction by inhibiting the phosphorylation of ERK, JNK, and p38, promote apoptosis of PASMCs, reduce oxidative stress and inflammatory responses, and alleviate vascular remodeling (Zhu et al., 2021). Jiedu Quyu Decoction, composed of *Carthamus tinctorius* L. (Honghua), *Reynoutria japonica* Houtt (Huzhang), *Pueraria montana* var. *lobata (Willd.) Maesen and S.M.Almeida ex Sanjappa* and *Predeep* (Gegen), *Allium chinense G. Don* (Xiebai), *Astragalus mongholicus Bunge* (Huangqi), can significantly reduce the mRNA and protein expression levels of NLRP3, caspase-1, IL-1β, and IL-18 in right heart tissues, inhibit the activation of NLRP3 inflammasome, reduce cardiomyocyte damage and fibrosis mediated by inflammatory factors, and exert anti-PH and cardioprotective effects (Ma et al., 2023). Xinyang Tablet, containing components such as quercetin and ginsenoside, can significantly reduce the protein expression of pro-inflammatory factors TNF-α and IL-6 in right ventricular tissues. It reduces the apoptosis rate of cardiomyocytes by upregulating the anti-apoptotic protein Bcl-2 and downregulating the pro-apoptotic proteins Bax and caspase-3, thereby improving right ventricular and pulmonary vascular remodeling (Gao et al., 2022).

#### 3.1.5 Additional immunomodulatory agents

In addition to monomers and compound prescriptions, some other types of natural products also play roles in immune regulation of PH. Triflavones are new active components isolated from *Selaginella doederleinii Hieron*. Triflavones exert immunomodulatory effects by inhibiting inflammatory cell infiltration and the TGF-β1/PI3K/Akt signaling pathway. In a hypoxia-induced rat PH model, triflavones can reduce the total number of cells, macrophage and neutrophil infiltration in bronchoalveolar lavage fluid, downregulate the levels of TGF-β1 and p-PI3K/Akt, reverse hypoxia-induced excessive proliferation of vascular smooth muscle cells and promote their apoptosis (Chen L. et al., 2021). Blueberry Extract is extracted from freshly ripened blueberries (*Vaccinium myrtillus* L.) and can alleviate inflammatory responses through antioxidant effects. In the MCT-induced PAH rat model, Blueberry Extract can reduce total reactive oxygen species (ROS), NADPH oxidase activity and lipid oxidation in lung tissues, restore the expression of transcription factor Nrf2, activate downstream antioxidant enzymes, and at the same time correct the balance of endothelin receptors, reducing ET-1-mediated inflammatory vascular remodeling (Turck et al., 2020; Leite et al., 2025). *Carthamus tinctorius* L. (safflower), a medicinal plant of the Asteraceae family, exerts immunomodulatory effects by inhibiting the NLRP3 inflammasome. It inhibits the activation of the NLRP3 inflammasome, regulates the TNF signaling pathway and Th17 cell differentiation, and reduces the expression of pro-inflammatory factors IL-1β, IL-18 as well as pulmonary vascular remodeling-related factors MMP-2, MMP-9, Collagen one and Collagen 3, thereby alleviating PAH (Ding et al., 2024). Ethyl Acetate Extract of Sceptridium ternatum intervenes in PH by inhibiting the NF-κB p65 signaling pathway and downregulating the expression of α-SMA, thereby alleviating pulmonary vascular remodeling and inflammatory damage (Xin et al., 2020).

In summary, a diverse array of natural products demonstrates potent immunomodulatory effects in PH models, primarily by dampening the activation of innate immune cells, including macrophages and neutrophils, restoring the balance of adaptive immunity such as the Treg/Th17 axis, and disrupting pro-inflammatory cytokine networks, specifically NF-κB and NLRP3. These interventions effectively reduce vascular inflammation and remodeling, positioning immunomodulation as a viable therapeutic strategy for PH. However, a significant limitation across many studies is the reliance on *in vivo* phenotypic readouts without robust *in vitro* mechanistic validation, making it difficult to distinguish direct immunomodulatory effects from secondary consequences. For prevalent flavonoids like isorhamnetin and baicalin, their known potential as pan-assay interfering substances (PAINS) raises concerns about the specificity of reported multi-target effects, which may represent assay artifacts rather than genuine therapeutic mechanisms. Furthermore, the evidence for Ginsenoside Rh1 remains largely computational or lacks proper positive controls, undermining their validity. Future research must prioritize rigorous characterization of single entities’ key pharmacological parameters and immune-cell-specific validation for credible therapeutics.

### 3.2 Metabolic regulatory natural products

A variety of natural products focus on mitochondrial function and intervene in energy metabolism disorders and pathological processes of pulmonary hypertension by improving glucose metabolism, lipid metabolism, and amino acid metabolism, as well as restoring the tricarboxylic acid (TCA) cycle. As systematically cataloged in Supplementary Table 1, metabolic-regulating natural products demonstrated efficacy in both *in vivo* and *in vitro* models.

#### 3.2.1 Reversing glycolytic reprogramming

Tagitinin C is a natural product isolated from the leaves of *Helianthus annuus* L. Tagitinin C reverses the Warburg effect in PAH by inhibiting the HIF-1β/PDK1 pathway. Under hypoxic conditions, tagitinin C reduces the levels of PDK1 and HIF-1β in PASMCs in a dose-dependent manner, blocks the inhibition of pyruvate dehydrogenase (PDH), restores mitochondrial TCA, and reverses aerobic glycolysis (Arai et al., 2021).

Green tea polyphenols, a class of epicatechin derivatives, are the primary active constituents in green tea. Green tea polyphenols reduce the production of PDK1, restore mitochondrial metabolism, and inhibit abnormal proliferation and migration of HPASMCs by inhibiting AKT1 phosphorylation and downregulating hypoxia-inducible factor 1α (HIF1α) expression (Yang et al., 2024).

#### 3.2.2 Restoring mitochondrial metabolism

Rutin is a rutinoside, a flavonol glycoside found in *Ruta graveolens* L. Rutin intervenes in PAH by targeting protein kinase Cα (PKCα) to inhibit ferroptosis and regulating mitochondrial metabolism. Rutin can reverse hypoxia-induced morphological abnormalities such as mitochondrial membrane rupture and cristae reduction in PASMCs. It can also regulate the glutamic acid metabolic pathway in lung tissues of PAH rats, affect the levels of related metabolites such as glutamic acid and phosphocholine, and restore the balance of mitochondrial energy metabolism. Specifically, rutin can significantly upregulate the expression of ferritin heavy chain (FTH) and glutathione peroxidase 4 (GPX4), and downregulate the expression of acylCoA synthetase long-chain family member 4 (ACSL4), thereby inhibiting iron-dependent lipid peroxidation. Rutin reduces the levels of Fe^2+^ and malondialdehyde (MDA) in lung tissues, increases glutathione (GSH) content, alleviates oxidative stress damage, and inhibits the occurrence of ferroptosis (Che et al., 2024).

Chrysin is a flavonoid compound extracted from organisms such as honey and propolis. Chrysin upregulates fatty acid transport genes Cd36 and Cpt1b, reduces long-chain fatty acid accumulation, promotes mitochondrial fatty acid oxidation, and simultaneously restores the expression of genes related to mitochondrial oxidative phosphorylation and TCA, increases total adenylate concentration, improves energy production, and ameliorates right ventricular dysfunction in PAH rat models (Kobayashi et al., 2022).

Qiliqiangxin capsule exerts its effects by reversing glycolytic metabolic shift and regulating energy metabolism enzymes. 18FDG-PET shows that treatment with Qiliqiangxin capsule significantly reduces right ventricular glucose uptake in the MCT model group, suggesting a reversal of metabolism towards aerobic oxidation. Its mechanism is related to the upregulation of PGC-1α, a key regulatory factor for mitochondrial biogenesis and oxidative metabolism, which increases significantly after treatment. In addition, Qiliqiangxin capsule improves mitochondrial dysfunction, indirectly enhances oxidative phosphorylation capacity, and reduces dependence on glycolysis (Lu et al., 2020).

#### 3.2.3 Regulating lipid and amino acid metabolism

18β-glycyrrhetinic acid is a pentacyclic triterpenoid compound extracted from *Glycyrrhiza glabra* L. It regulates energy metabolism to improve glycolysis, gluconeogenesis, and the TCA cycle by increasing the activities of superoxide dismutase (SOD) and glutathione peroxidase (GSH-Px), while reducing MDA. Additionally, 18β-glycyrrhetinic acid can correct metabolic disorders of branched-chain amino acids (BCAAs) such as leucine and isoleucine, downregulate BCAA levels, and upregulate proline and citrulline, thereby alleviating vascular fibrosis. It also improves the accumulation of lipid metabolism intermediates (ketone bodies) and low-density lipoprotein, involving 10 key metabolic pathways such as various amino acid biosynthesis and pyruvate metabolism, reversing the metabolic network disorders caused by HAPH at the systemic level (Yang et al., 2021).

Kaempferol is a natural flavonoid isolated from *Delphinium*. Kaempferol reverses the abnormal elevation of TCA cycle-related metabolites such as succinic acid and citric acid in the plasma of PAH rats by regulating amino acid metabolism and arachidonic acid metabolism, downregulates the pro-inflammatory metabolite 12-HETE, upregulates the anti-inflammatory metabolite DHA, and inhibits the expression of PTGS1 (Yi et al., 2022).

Osthole is a coumarin compound extracted from *Cnidium monnieri*. Osthole regulates microRNA-22-3p-mediated lipid metabolic reprogramming, reduces serum total cholesterol and triglyceride/high-density lipoprotein cholesterol ratio, inhibits fatty acid transporters such as CD36 and fatty acid synthase (FAS), decreases fatty acid uptake, synthesis, and oxidation, and alleviates pulmonary vascular remodeling in PAH (Niu et al., 2022).

Luteolin is also a flavonoid compound. It downregulates the gene and protein expression of COX1, 5-LOX, 12-LOX, and 15-LOX in the arachidonic acid pathway, restores the levels of abnormal metabolites such as PGE2, PGD2, LTB4, 12-HETE, 15-HETE, PGF2α, and 8-iso-PGF2α in PAH models, inhibits abnormal proliferation of PASMCs, and reverses pulmonary vascular remodeling (Song et al., 2022).

#### 3.2.4 Targeting arginine-NO pathway

L-citrulline, a precursor of L-arginine, is a natural amino acid compound. L-citrulline significantly increases plasma arginine levels, restores the substrate availability of endothelial nitric oxide synthase (eNOS), and promotes enzyme coupling. Combined with tetrahydrobiopterin (BH_4_) treatment, it can reduce superoxide production in the pulmonary artery and increase NO production, thereby decreasing PVR and improving vascular function (Dikalova et al., 2020). Low concentrations of L-citrulline induce the expression of arginase II, while high concentrations combined with arginase inhibitors can further enhance the recovery of eNOS uncoupling and NO production, showing dose-dependent metabolic regulation (Douglass et al., 2021).

Vanillic Acid is a phenolic compound widely present in edible plants and fruits in nature. Vanillic Acid inhibits arginase activity and the expression of Hif-2α/Hif-1β, promotes the expression and phosphorylation of eNOS, enhances the NO signaling pathway, and alleviates pulmonary vasoconstriction, remodeling, and fibrosis in PAH (Wang et al., 2022).

Hawthorn flavonoid extract is an extract from plants of the *Crataegus oxyacantha* genus with flavonoids as the main active components. Hawthorn flavonoid extract protects vascular endothelium by scavenging oxygen free radicals, activates eNOS to promote NO production and mediate vasodilation, inhibits Ca^2+^-ATPase to regulate intracellular calcium homeostasis, and reduces pulmonary artery pressure (Ahmadipour et al., 2020). It can also promote body growth and metabolism, alleviate cardiac hypertrophy, decrease the ratio of right ventricular weight to total ventricular weight, and optimize cardiac metabolic function.

#### 3.2.5 Additional metabolic agents

Some other types of natural products also play roles in metabolic regulation of PH. Rhodiola crenulata extract is a complex extracted from plants of the *Rhodiola* genus, mainly containing flavonoids and their glycosides. Rhodiola crenulata extract functions by inhibiting fatty acid oxidation (FAO) and regulating phospholipid metabolism. It targets acylcarnitines, reduces abnormally elevated acylcarnitine levels in serum, and inhibits the mRNA and protein expression of carnitine palmitoyltransferase 1A (CPT1A) (Ren et al., 2021). Lipidomics shows that Rhodiola crenulata extract can reverse the imbalance of phosphatidylcholine and acylcarnitines in the serum of PAH rats and inhibit FAO-driven metabolic reprogramming. In addition, Rhodiola crenulata extract downregulates autophagy markers LC3B and ATG7, upregulates p62, blocks the LKB1-AMPK pathway, and reduces autophagy-mediated lipid breakdown for energy supply, thereby improving PAH (Ren et al., 2021).

This section highlights the emerging role of natural products in counteracting the pathological metabolic reprogramming characteristic of PH. The discussed compounds, such as Tagitinin C, Green tea polyphenols, Rutin, and 18β-glycyrrhetinic acid, target various metabolic facets. Their actions include reversing the Warburg effect, restoring mitochondrial oxidative metabolism, regulating lipid and amino acid homeostasis, and enhancing the arginine-NO pathway. Agents like Qiliqiangxin capsule demonstrate the ability to shift cellular energy production back towards oxidative phosphorylation *in vivo*. Nevertheless, some compounds, including 18β-glycyrrhetinic acid and luteolin, are reported to modulate broad metabolic networks, but these findings often derive from untargeted metabolomics in complex *in vivo* systems, failing to pinpoint primary molecular targets or establish causal relationships. The reliance on phenotypic assays, without genetic or chemical target validation, means that the proposed mechanisms for Tagitinin C or green tea polyphenols remain speculative. Moreover, the relevance of reversing the Warburg effect in the context of PH vasculature requires deeper exploration in human-derived cells and tissues. To advance, future studies should focus on delineating specific on-target effects using validated chemical probes, conducting rigorous ADME (Absorption, Distribution, Metabolism, Excretion) studies, and prioritizing agents with favorable therapeutic indices for preclinical development.

### 3.3 Immune-metabolic dual-regulating natural products

#### 3.3.1 Monomers targeting immune-metabolic hubs

Monomeric components in immune-metabolic dual-regulating natural products exhibit synergistic intervention effects in PH treatment by simultaneously acting on immune-inflammatory pathways and key targets of metabolic remodeling.

Astragaloside IV, the main active component of *Astragalus membranaceus*, belongs to pentacyclic triterpenoids. Astragaloside IV is a typical representative of the interactive regulation between immune cell differentiation and metabolic pathways, exerting dual effects by regulating T cell subset differentiation and metabolic pathways. Astragaloside IV can inhibit the phosphorylation of the mTOR signaling pathway, downregulate the Bcl-6 transcription factor in Tfh, and upregulate the CTLA-4 expression in T follicular regulatory cells (Tfr), thereby reshaping the Tfh/Tfr balance to reduce the release of pro-inflammatory factors such as IL-21. Meanwhile, it inhibits the NF-κB-mediated inflammatory cascade to regulate the immune microenvironment (Li et al., 2022). In terms of metabolic pathways, ASIV upregulates prolyl hydroxylase 2 (PHD2) to promote the degradation of HIF1α, blocks the HIF1α/NLRP3 inflammasome pathway to inhibit pyroptosis of PASMCs, and downregulates the transcription of the downstream fibrotic factor Collagen I of HIF1α (Jin et al., 2021; XinTian et al., 2022; Xi et al., 2023). Astragaloside IV inhibits the mTOR/RhoA signal, upregulates p27 to arrest the cell cycle of PASMCs, and synergistically inhibits the proliferation and migration of vascular smooth muscle. Ultimately, through the interactive regulation of core targets such as mTOR-PHD2/HIF1α-RhoA/p27, it achieves multiple effects including anti-inflammation, anti-fibrosis, and inhibition of vascular remodeling.

Quercetin is a flavonol widely distributed in plants. Quercetin can block the HMGB1/RAGE/NF-κB axis to inhibit the release of pro-inflammatory factors such as TNF-α and IL-6, target the NLRP3 inflammasome to block the maturation of IL-1β/IL-18, and restore the balance of immune cell apoptosis by upregulating miR-204 to inhibit the HIF1α/NFATc2 axis and the Src-STAT3-NFAT pathway (Rajabi et al., 2020; Zhang N. et al., 2023; Ding et al., 2024; Kordestani et al., 2024). At the metabolic level, as a mixed inhibitor of CYP1A1 and a strong inhibitor of UGT1A9, quercetin increases the systemic exposure of riociguat (Li Q. et al., 2024). Meanwhile, it inhibits the phosphorylation of MAPK1 (ERK1/2) to block the proliferation of PASMCs, downregulates NOX4 to reduce ROS production for alleviating oxidative stress, and improves right ventricular fibrosis and energy metabolism by regulating the TGF-β1/Bcl-2-Bax axis (Luo et al., 2021; Rajabi et al., 2021). The NF-κB and NOX4 pathways form a bidirectional regulatory network, constituting an immune-metabolic synergistic effect, and multi-targetedly intervene in NLRP3, CYP1A1, NOX4, and PD-1/PD-L1 immune checkpoints to reconstruct the metabolic homeostasis of lung tissues (Luo et al., 2021; Zhang N. et al., 2023; Ding et al., 2024; Li Q. et al., 2024).

Berberine is an isoquinoline alkaloid in the dried rhizomes of plants belonging to the genus *Coptis* (Ranunculaceae), with similar effects to quercetin (Luo et al., 2021). Berberine inhibits the nuclear translocation of β-catenin and the expression of target genes such as cyclin D1 and VEGF by downregulating Trx1, and simultaneously inhibits MAPK1 (ERK1/2) phosphorylation and NOX4-mediated ROS production, thereby blocking the metabolic reprogramming of abnormal PASMC proliferation (Yu et al., 2020; Luo et al., 2021). In addition, Berberine reduces the release of inflammatory factors such as TNF-α and IL-6 by inhibiting the CYP1B1 and NF-κB pathways, upregulates miR-204 to target and regulate HIF1α/NFATc2, inhibits the crosstalk between oxidative stress and inflammation, and suppresses abnormal cell apoptosis and fibrosis by regulating the Bax/Bcl-2 ratio and TGF-β1 expression (Luo et al., 2021; Rajabi et al., 2021; Kordestani et al., 2024). Through the synergistic effects of pathways including Trx1/β-catenin, MAPK1/NOX4, and miR-204/Src-STAT3, it achieves dual regulation of cellular metabolic homeostasis and immune-inflammatory networks, improving pulmonary vascular remodeling and right ventricular function.

Resveratrol is a plant polyphenol found in high concentrations in *Veratrum album* L. Resveratrol inhibits STAT3 phosphorylation to reduce Th17 cell differentiation, downregulates inflammatory factors such as IL-6 and VCAM-1, activates Nrf2/Trx-1 to exert antioxidant effects, promotes LC3-II-mediated autophagy, inhibits vWF/P-selectin-dependent platelet activation, and blocks TGF-β/Smad3 to alleviate fibrosis (Li et al., 2020; Vazquez-Garza et al., 2020; Liu X. et al., 2022). Meanwhile, resveratrol regulates the metabolism of leucine and tryptophan as well as metabolites such as hydroxyphenyllactic acid and isopalmitic acid, restores the level of citric acid in the TCA cycle, regulates fatty acid metabolism through the PPAR pathway, and integrates deacetylation with SIRT1 as the hub, thereby synchronously improving mitochondrial function and the immune-inflammatory network, and inhibiting pulmonary vascular remodeling and right ventricular damage (Chen Y. et al., 2020; Vazquez-Garza et al., 2020; Sun et al., 2021; Liu X. et al., 2022).

Scutellarein is a natural flavonoid compound. It mediates the deacetylation of nicotinamide nucleotide transhydrogenase (NNT) by activating Sirtuin 1 (SIRT1), enhances the catalytic activity of NNT to increase NAD^+^ levels, and forms a SIRT1/NNT/NAD^+^ positive feedback loop, thereby maintaining mitochondrial metabolic homeostasis. Meanwhile, it inhibits NLRP3 inflammasome-related immune inflammation and pyroptosis of PASMCs, improving vascular remodeling in PH (Tang et al., 2025).

Ginsenoside Rg1 is a ginsenoside found in *P. ginseng C.A.Mey*. Ginsenoside Rg1 downregulates calpain-1 to inhibit STAT3 phosphorylation, blocks the IL-6/STAT3 inflammatory pathway and the release of inflammatory factors, and can inhibit the activation of the TXNIP/NLRP3 inflammasome, reducing IL-1β secretion (Zhang R. et al., 2025). Additionally, Ginsenoside Rg1 reduces mitochondrial oxidative stress by inhibiting TXNIP/NLRP3, downregulates mitophagy proteins such as PINK1/Parkin to maintain mitochondrial homeostasis, and simultaneously increases the activity of antioxidant enzymes and enhances the eNOS/NO pathway to improve endothelial function and pulmonary vascular remodeling (Ran et al., 2024; Zhang R. et al., 2025).

2-phenylethyl-beta-glucopyranoside is a glycoside compound derived from the dried root tubers of *Rehmannia glutinosa*, a herbaceous plant of the Scrophulariaceae family. 2-phenylethyl-beta-glucopyranoside regulates oxidative stress metabolism by inhibiting the PI3K/Akt/mTOR pathway, reducing ROS and MDA levels in lung tissues, and increasing SOD and GSH-Px activities. Meanwhile, it reduces the number of myeloid-derived suppressor cells and regulatory T cells, and increases the number of immune cells such as natural killer cells, thereby achieving dual regulation of immunity and metabolism in hypoxic pulmonary hypertension (Zeng et al., 2024).

Hydroxysafflor yellow A is a polyphenolic compound extracted from the traditional Chinese medicine *C. tinctorius* L. (safflower). Hydroxysafflor yellow A targets inflammation-related proteins such as ANXA5 and SRC, and activates PPARG to regulate lipid metabolism and oxidative stress responses. As a hub target, PPARG integrates anti-inflammatory and metabolic regulatory functions, improving vascular remodeling and right ventricular hypertrophy in PAH rats (Ji et al., 2024).

#### 3.3.2 Compound formulations with synergistic regulation

Compound natural products exert synergistic effects in the immune-metabolic interaction network. Lingguizhugan Decoction, composed of *Poria cocos(Schw.)Wolf* (Fuling), *Neolitsea cassia* (L.) Kosterm (Guizhi), *Atractylodes macrocephala Koidz.* (Baizhu), and *G. glabra* L (Gancao), can inhibit the CCL2/CXCR4 axis to reduce M2 macrophage infiltration, while downregulating HMOX1/NOX4 to inhibit ferroptosis, and regulating the PPAR signaling pathway and fatty acid metabolism, thus forming immune-metabolic synergistic regulation (Shi et al., 2024). Zhishi-Xiebai-Guizhi Decoction is composed of *A. chinense* G. Don (Xiebai), Trichosanthes kirilowii Maxim (Gualou), *Citrus* × *aurantium f. aurantium* (Zhishi), *Magnolia officinalis Rehder and E.H.Wilson* (Houpo), and *N. cassia (L.) Kosterm.* (Guizhi). The active components of Zhishi-Xiebai-Guizhi Decoction inhibit immune-inflammatory factors such as IL-6 and TNF, and simultaneously inhibit the HIF-1α/PI3K/Akt signaling pathway to reverse glycolytic reprogramming. As a hub molecule, HIF-1α regulates the balance of IL-6/IL-10 and PLIN2/toxic lipid metabolism, achieving dual phenotypic improvement (Fu et al., 2024; Huang et al., 2024). Shufeiya Recipe contains components such as quercetin and luteolin. Shufeiya Recipe inhibits oxidative stress by activating SIRT3/FOXO3a to upregulate Mn-SOD and COX-1/2, and simultaneously activates PI3K/AKT/eNOS to promote NO/sGC/cGMP/PKG-mediated vasodilation, while inhibiting the Ras/MEK1/2/ERK1/2/c-fos pathway to reduce cell proliferation, thereby regulating oxidative stress and vascular remodeling (Jia et al., 2022; Zhang et al., 2022). Qishen Yiqi Formula is a traditional Chinese medicine compound, which is a mixture of extracts from four Chinese medicines: *A. mongholicus Bunge*, *Salvia miltiorrhiza Bunge*, *Panax notoginseng (Burkill) F.H.Chen*, and *Dalbergia odorifera T.C.Chen*. Qishen Yiqi Formula not only inhibits the NF-κB pathway to reduce inflammatory responses, but also regulates metabolic reprogramming of PASMCs through the HIF-1 signaling pathway to inhibit glycolysis-related proliferation. The core gene TP53 further embodies dual effects by arresting the cell cycle (Wu et al., 2021).

#### 3.3.3 Additional dual-function agents

Other types of compounds also exhibit synergistic effects in inhibiting inflammatory responses, regulating cellular metabolism, and improving vascular remodeling in PH. The extract of Salvia przewalskii is a mixture containing various phenolic acids and terpenoids. The extract of Salvia przewalskii regulates immune inflammation, inhibits abnormal cell proliferation and vascular remodeling by downregulating HIF-1α, PCNA, Bcl-2, CDK4, CyclinD1, P27Kip1, and inhibiting pathways such as RhoA-ROCK, MCP-1, NF-κB as well as pro-inflammatory factors. It can also increase the activities of SOD and LDH, reduce MDA levels, regulate oxidative stress metabolism, and improve PH (Wang et al., 2020). Grape seed procyanidin is a polyphenolic flavonoid compound extracted from *Vitis vinifera* L., which integrates immune and metabolic regulation through the PPARγ/COX-2 pathway. Grape seed procyanidin activates peroxisome proliferator-activated receptor γ (PPARγ), thereby downregulating the expression of cyclooxygenase 2 (COX-2), achieving the regulation of lipid metabolism, inhibiting cell proliferation, blocking the inflammatory cascade, alleviating vascular inflammation, and reversing the vascular structural remodeling of PASMCs (Liu et al., 2020). Aureane-type sesquiterpene tetraketides, a class of compounds isolated from the fungus *Myrothecium gramineum*, are novel immunomodulators with IL-17A inhibitory activity. Aureane-type sesquiterpene tetraketides inhibit IL-17A secretion by downregulating the transcription factor RORγt, and target and inhibit the glycolysis and gluconeogenesis pathways to block energy metabolism in Th17 cells, thereby improving inflammatory responses and vascular smooth muscle cell proliferation in PH (Tang et al., 2023).

The pharmacological profiles of these dual-regulating monomers, including their molecular targets, effective doses, and experimental models, are systematically summarized in Supplementary Table 1. These natural products exert synergistic effects including immune-metabolic crosstalk, mitochondrial-inflammasome integration, inhibiting inflammation and oxidative stress, regulating metabolism, and achieving anti-inflammatory, anti-fibrotic, anti-vascular remodeling outcomes. Monomeric compounds such as Astragaloside IV and Quercetin precisely intervene in the mTOR/NF-κB/NLRP3 axis, while compound preparations such as Lingguizhugan Decoction and Qishen Yiqi Formula strengthen the regulation of immune-metabolic networks through the synergistic effect of multiple components, providing new strategies for the development of natural drugs for PH treatment. While Supplementary Table 1 lists impressive multi-target effects for these compounds, the experimental designs often do not conclusively prove that the immunomodulatory and metabolic effects are mechanistically linked rather than parallel, independent events. For the compound formulations, the synergistic effects are postulated based on network pharmacology or limited experimental models, but without decomposing the mixtures to identify key active constituents and their interactions, these claims remain largely theoretical. The field should adopt more rigorous standards, including the use of selective inhibitors and genetic tools to validate proposed hubs, and employ advanced *ex vivo* models like patient-derived cells or organoids to better assess the therapeutic relevance of these dual-targeting approaches before considering clinical translation.

**FIGURE 5:**
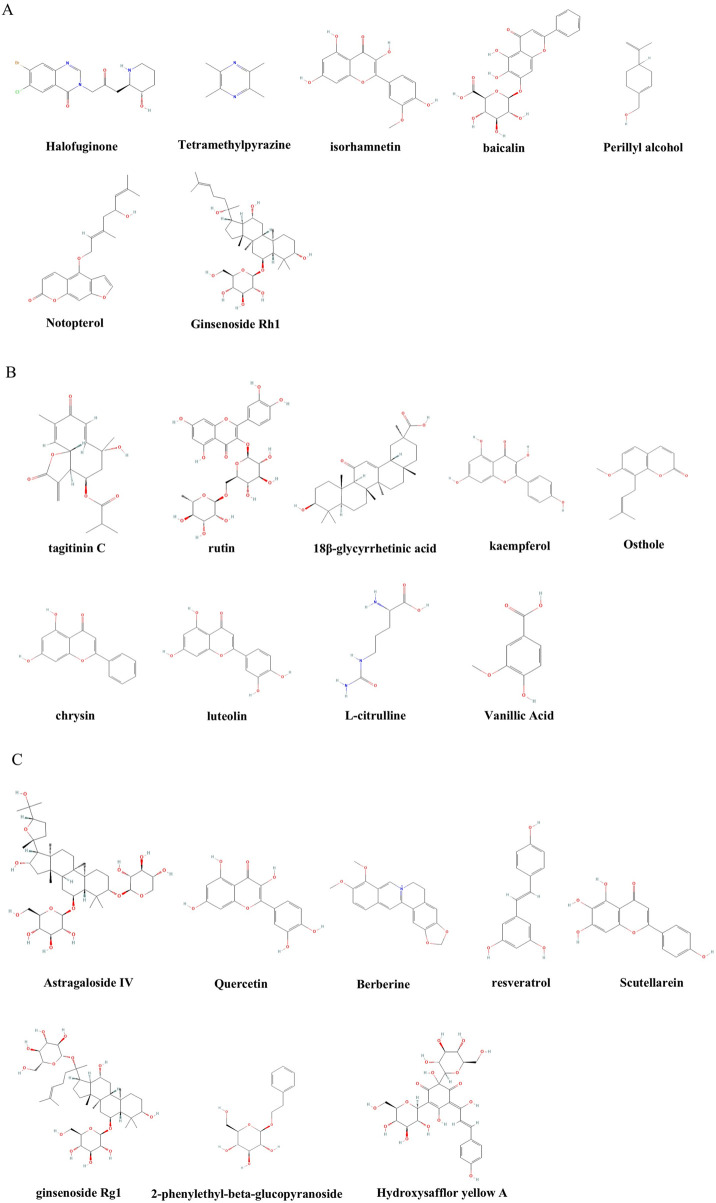
Chemical structures of the natural products discussed in this review. **(A)** Immunomodulatory natural products **(B)** Metabolic regulatory natural products. **(C)** Immune metabolic dual regulatory natural products. Structure obtained from PubChem (https://pubchem.ncbi.nlm.nih.gov/).

The chemical structures of the representative immunomodulatory, metabolic regulatory, and immune-metabolic dual-regulating natural products discussed in this section are summarized in Figure 5.

## 4 Discussion

The core pathological mechanism of PH lies in immune system dysfunction and metabolic remodeling, which together form an immune-metabolic interactive regulatory network. This review systematically elaborates on key links in this network, including innate/adaptive immune imbalance, inflammatory factor storm, multi-cellular metabolic reprogramming, abnormal key metabolites, and epigenetic regulation, with a focus on sorting out natural product intervention strategies targeting this network.

Immune imbalance inhibits mitochondrial oxidative phosphorylation and activates glycolysis through pro-inflammatory factors such as IL-1β and IL-6, as well as NLRP3 inflammasome signaling molecules, resulting in metabolic remodeling. Meanwhile, intermediate products such as lactic acid and succinic acid generated by metabolic reprogramming, along with reactive oxygen species (ROS), reversely activate immune-inflammatory pathways such as NLRP3 and cGAS-STING, forming an immune-metabolic vicious cycle. Epigenetic regulatory layers such as DNA methylation, histone modification, and non-coding RNA act as key hubs to integrate immune stimulation and metabolic stress signals, coordinating pathological gene expression. Given the limited effect of single intervention, the multi-target intervention of natural products can synergistically regulate immune-metabolic imbalance, showing greater therapeutic potential.

Natural products intervene in the immune-metabolic network through multi-pathway integration mechanisms, demonstrating unique advantages in PH treatment. Immunomodulatory natural products improve the immune microenvironment to indirectly alleviate metabolic stress by inhibiting core inflammatory pathways such as NF-κB and MAPK, and regulating immune cell infiltration. Metabolic-regulating natural products focus on restoring mitochondrial oxidative phosphorylation function and correcting abnormalities in glucose, lipid, and amino acid metabolism, providing a metabolic basis for the reconstruction of immune homeostasis. Dual-regulating natural products synchronously regulate immune responses and metabolic activities by targeting hub molecules such as mTOR and SIRT1: they synchronously inhibit Tfh cell differentiation and HIF-1α-mediated glycolysis, or form anti-inflammatory-metabolic protective synergistic effects by intervening in the TXNIP/NLRP3 inflammatory axis and PINK1/Parkin mitochondrial homeostasis, thereby effectively blocking the vicious cycle of immune activation-metabolic disorder-vascular remodeling. This multi-component and multi-target systematic intervention model precisely matches the multi-dimensional pathological characteristics of the immune-metabolic network. Ultimately, by inhibiting abnormal proliferation of PASMCs and endothelial cells, reducing extracellular matrix deposition, and improving myocardial energy metabolism patterns, it achieves the therapeutic goal of delaying pulmonary vascular remodeling and the progression of right heart failure, providing a unique multi-dimensional regulatory strategy of natural drugs for PH treatment.

Current research on the regulation of PH by natural products, while promising, is fraught with significant limitations that challenge the validity and translational potential of the findings. Firstly, there is insufficient depth and integration in mechanism analysis. The overreliance on phenotypic observations in simplistic *in vitro* systems is a major concern. For well-known PAINS like quercetin and luteolin, many reported multi-target effects must be interpreted with extreme caution, as they may represent non-specific binding or assay artifacts rather than genuine therapeutic mechanisms. Existing studies mostly focus on single pathways or cell types, lacking a comprehensive explanation of the systemic remodeling of immune-metabolic networks, especially the mechanisms of inter-organ metabolic-immune axes and metabolic crosstalk among different immune cell subsets remain unclear (Thenappan and Weir, 2024; Yang et al., 2025). The identification of direct targets and upstream signaling pathways still predominantly relies on computational prediction or correlative data rather than direct experimental validation, such as using chemical biology probes or genetic knockout models, leading to a high risk of false-positive claims (Wang et al., 2024). Although epigenetic regulatory mechanisms have been confirmed as key intervention links, relevant research is still weak, and it is necessary to strengthen studies on the role of non-coding RNAs such as circRNA, lncRNA, and miRNA, as well as DNA/histone modifications in mediating the effects of natural products (Gamen et al., 2016; Chen B. et al., 2025; Hu et al., 2025). Secondly, the evidence base is predominantly pre-clinical and suffers from poor generalizability to human disease. The vast majority of evidence comes from MCT or hypoxia animal models, whose pathological characteristics differ from human PH, lacking in-depth verification in models that are more similar to human disease states. Furthermore, some pharmacological studies fail to report essential details such as minimal active concentrations, full dose-response relationships, the use of appropriate positive and negative controls, and proper characterization of the natural material used, including taxonomic validation and chemical standardization. This omission makes it impossible to critically assess the robustness of the original findings. The clinical translation process is slow; except for a few compound preparations such as Qiliqiangxin capsule, most natural products lack rigorous randomized controlled trials (RCT) to evaluate their efficacy and safety. In addition, the complexity of compound components leads to unclear pharmacodynamic material basis and multi-component interaction mechanisms, which need to be analyzed using spectrum-effect relationship technology. Some existing studies have shown that components such as flavonoids and saponins generally have pharmacokinetic defects such as low oral bioavailability and rapid metabolism, and the optimization of administration regimens and targeted delivery systems such as nano-formulations is still in the exploratory stage (Provencher et al., 2018; Fu et al., 2023; Zhang J.-j. et al., 2024).

To promote the development of natural products in the regulation and treatment of the immune-metabolic network in PAH, future research needs to advance synergistically from three dimensions: mechanism analysis, clinical translation, and technological breakthroughs. Firstly, by combining chemical biology techniques such as target fishing and molecular probes with single-cell multi-omics technologies, dynamically analyze the systemic remodeling of immune cell metabolic phenotypes, epigenetic modifications, and metabolite networks in lung tissues and the circulatory system after natural product intervention. Crucially, proposed targets and pathways must be validated using orthogonal methods. Emphasis should be placed on clarifying the regulatory pathways of immune-metabolic axes mediated by dual-regulating molecules through hub molecules such as mTOR and HIF-1α, and expanding mechanistic exploration in emerging fields such as the gut-lung axis and neuro-immune-metabolic crosstalk (Harbaum et al., 2021; Chen et al., 2024; Lv et al., 2024). Secondly, the process of clinical translation should be accelerated. Future efforts must prioritize the quality of pre-clinical data. Candidate molecules should be selected based on robust, reproducible evidence from well-controlled studies that adequately address PAINS concerns and demonstrate target engagement. Standardized preclinical evaluations, and high-quality RCTs should be designed to assess their efficacy and safety in PH patients, with a focus on populations with poor responses to existing therapies. Meanwhile, explore strategies for synergistic drug use with endothelin receptor antagonists and prostacyclin analogs (Alencar et al., 2017; Vaidya et al., 2017). In addition, it is necessary to clarify the pharmacodynamic material basis of compound preparations through spectrum-effect relationship analysis, systematically conduct pharmacokinetic studies to optimize absorption and distribution characteristics, and utilize nanotechnology, such as metal-organic framework carriers and exosome biomimetic delivery, to develop intelligent delivery systems targeting pulmonary blood vessels and immune cells. Combined with optimization of preparation processes, stable and controllable release of active ingredients can be achieved. Finally, and critically, the field must adopt higher standards of reporting, including full chemical and taxonomic characterization of natural products, detailed pharmacological data, and stringent assessment of data validity to minimize false positives and enhance reproducibility. Ultimately, a complete innovation chain from molecular mechanisms to clinical applications should be constructed, providing scientific support for the development of PH systemic intervention strategies based on natural products (Yong et al., 2024).

## 5 Conclusion

The immune-metabolic interactive regulatory network is the core driver of the occurrence and development of PH, providing new perspectives and targets for treatment. Natural products, especially monomers and compound preparations with immune-metabolic dual regulatory activities, have shown unique value in regulating this network, reversing pulmonary vascular remodeling, and improving right heart dysfunction by virtue of their advantages in multi-target synergistic intervention. However, current research faces significant challenges in terms of mechanism depth, model relevance, and clinical translation. Future studies need to deeply integrate cutting-edge technologies to deepen mechanism analysis, focus on breaking through dual regulatory mechanisms, and vigorously promote rigorous clinical evaluation and the development of new formulations, so as to provide more effective and safer treatment options for patients.
