# Supplementary material for: Natural products targeting the immune-metabolic regulatory network in pulmonary hypertension: mechanisms, classification, and therapeutic prospects
Source: Front Pharmacol. 2026 Jan 8;16:1712903. doi: 10.3389/fphar.2025.1712903 (PMC12824024; doi:10.3389/fphar.2025.1712903)
Supplement: Supplementary file 1 [file Table1.docx]

**Table S1 Therapeutic mechanisms of active components from natural medicines in Pulmonary Hypertension**

| **Natural medicines** | **Molecular formula** | **Categories** | **Sources** | **Study type** | **model** | **Controls** | **Minimal effective concentration / Optimal dose** | **duration** | **Mechanism** | **Reference** |
| --- | --- | --- | --- | --- | --- | --- | --- | --- | --- | --- |
| **Halofuginone** | C_16_H_17_BrClN_3_O_3_ | Quinazolinone alkaloid derivative | Hydrangea febrifuga (Lour.) Y.De Smet & Granados [Hydrangeaceae] | In vivo | Male SD rats with HAPH induced by exposure to a simulated 6000 m hypoxic environment | distilled water | In vivo: 1 mg/kg | 4 weeks | ↓TGF-β1, ↓ Smad2/3, IL-1β↓, IL-6↓, TNF-α ↓, ↓RVHI, ↓PVWT. | (Wang et al., 2025) |
| **Tetramethylpyrazine** | C_8_H_12_N_2_ | Amide alkaloid | Conioselinum anthriscoides 'Chuanxiong' [Apiaceae] | In vivo  In vitro | 1. MCT-induced PAH rat model  2. Primary cultured rat PASMCs | 0.9% NaCl solution | In vivo: 100 mg/kg | Not mentioned | ↓ HMGB1, ↓PERK/ATF4, ↓ SIAH2, ↑HIPK2, IL-1β↓, IL-6↓. | (Zhang et al., 2023b) |
| **Isorhamnetin** | C_16_H_12_O_7_ | Flavonoids | Hippophae rhamnoides L. [Elaeagnaceae] | In vivo  In vitro | 1. MCT-induced PAH rat model  2. TNF-α-induced human PASMCs proliferation model | sildenafil | In vivo: 100 mg/kg and 150 mg/kg  In vitro: 100 μm | In vivo: 21 days In vitro: 24 h | ↑BMPR2/Smad, ↓TNF-α, ↓IL-6, ↓ PASMC proliferation | (Chang et al., 2020) |
| **baicalin** | C_21_H_18_O_11_ | Flavones | Scutellaria baicalensis Georgi [Lamiaceae] | In vivo  In vitro | 1. MCT-induced PAH rat model  2. TNF-α-induced rat PASMCs proliferation model | saline solution | In vivo: 100 mg/kg  In vitro: 100 μg/ml | In vivo: 4 weeks In vitro: 24-72 h | ↑BMPR2/Smad, ↓IL-6, ↓TNF-α/NF-κB, ↓ PASMC proliferation | (Xue et al., 2021) |
| **Perillyl alcohol** | C_10_H_16_O | Monoterpene | Perilla frutescens (L.) Britton [Lamiaceae] | In vivo | MCT-induced PAH rat model | Saline；  vehicle (0.5 mL 5% ethanol) | In vivo: 50 mg/kg | 3 weeks | miR-204, ↓Src–STAT3–NFAT, ↓Inflammatory Cell Infiltration, Apoptosis ↔ Cell Proliferation, Anti-Oxidative Stress. | (Rajabi et al., 2020; Kordestani et al., 2024) |
| **Notopterol** | C_21_H_22_O_5_ | Linear furocoumarin | Hansenia weberbaueriana (Fedde ex H.Wolff) Pimenov & Kljuykov [Apiaceae] | In vivo  In vitro | 1. MCT-induced PAH rat model  2. Human PASMCs under hypoxia | vehicle [1% DMSO +10% Tween 80+0.5%sodium cellulose +89% saline] | In vivo: 20 mg/kg  In vitro: 10 μm and 20 μm | In vivo: 3 weeks  In vitro: 36 h | ↓NF-κB, ↓IL-1β, ↓IL-6, ↓Macrophage. | (Huang et al., 2022) |
| **Ginsenoside Rh1** | C_36_H_62_O_9_ | Tetracyclic triterpenoid | Panax ginseng C.A.Mey. [Araliaceae] |  |  |  |  |  | →ITGAV, ITGA4, ITGA9, THBS1, ITGA2, COL4A3, ↓Treg, ↓Th1, ↓mast cells, ↓macrophages. | (Li and Zhang, 2023) |
| **Buyang Huanwu Decoction** |  |  | Astragalus mongholicus Bunge [Fabaceae]  Angelica sinensis (Oliv.) Diels [Apiaceae]  Paeonia lactiflora Pall. [Paeoniaceae]  Conioselinum anthriscoides 'Chuanxiong' [Apiaceae]  Juglans regia L. [Juglandaceae]  Carthamus tinctorius L. [Asteraceae]  Dilong (Pheretima) | In vivo | MCT-induced PH model in SD rats | sildenafil | In vivo: 20 g/kg | 21 days | ↑PI3K-Akt-eNOS, ↑ NO, ↓PASMC proliferation and migration. ↓TNF, ↓inflammation. | (Chen et al., 2021b) |
| **Xinmai 'an** |  |  | Panax ginseng C.A.Mey. [Araliaceae]  Astragalus mongholicus Bunge [Fabaceae]  Salvia miltiorrhiza Bunge [Lamiaceae]  Paeonia lactiflora Pall. [Paeoniaceae]  Ophiopogon japonicus (Thunb.) Ker Gawl. [Asparagaceae] Blumea balsamifera (L.) DC. [Asteraceae] | In vivo  In vitro | 1. MCT-induced PAH model in SD rats 2.PASMCs from rats | sildenafil | In vivo: 251.6 mg/kg In vitro: 250 μg/mL | In vivo: 2 weeks  In vitro: 48 h | ↓Phosphorylation of ERK, JNK, and p38, ↓MAPK. ↑PASMC apoptosis, ↓OS, ↓inflammatory, ↓Vascular remodeling. | (Zhu et al., 2021) |
| **Jiedu Quyu Decoction** |  |  | Carthamus tinctorius L. [Asteraceae]  Reynoutria japonica Houtt. [Polygonaceae]  Pueraria montana var. lobata (Willd.) Maesen & S.M.Almeida ex Sanjappa & Predeep [Fabaceae]  Allium chinense G.Don [Amaryllidaceae]  Astragalus mongholicus Bunge [Fabaceae] | In vivo | MCT-induced right-sided heart failure associated with PAH in SD rats | furosemide | In vivo:12.6 g/kg | 14 days | ↓MRNA and protein expression levels of NLRP3, caspase-1, IL-1 β, IL-18, ↓NLRP3, ↓Inflammatory factors, ↓myocardial cell damage and fibrosis. | (Ma et al., 2023) |
| **Xinyang Tablet** |  |  | Astragalus mongholicus Bunge [Fabaceae]  Epimedium sagittatum (Siebold & Zucc.) Maxim. [Berberidaceae]  Panax ginseng C.A.Mey. [Araliaceae]  Leonurus japonicus Houtt. [Lamiaceae]  Ilex pubescens Hook. & Arn. [Aquifoliaceae]  Descurainia sophia (L.) Webb ex Prantl [Brassicaceae]  Plantago asiatica L. [Plantaginaceae] | In vivo  In vitro | 1. SD rats with chronic hypoxia-induced right ventricular remodeling  2. H9c2 cells induced by CoCl_2_ to establish hypoxia injury model | Captopril | In vivo: 270 mg/kg  In vitro: 30 μg/mL and 60 μg/mL | In vivo: 3 weeks  In vitro: 24 h | ↓TNF-α, ↓IL-6, ↑Bcl-2, ↓Bax, ↓caspase-3, ↓CMAR. | (Gao et al., 2022) |
| **Triflavones** |  | Flavones | Selaginella doederleinii Hieron. [Selaginellaceae] | In vivo  In vitro | 1. Hypoxia-induced PH rat model 2. Hypoxia-induced rat aortic smooth muscle cells (ASMCs) model | 0.9% normal saline | In vivo: 100 mg/kg  In vitro: 100 μg/mL | In vivo: 3 weeks  In vitro: 24 h | ↓TGF-β1/PI3K/Akt, ↓Total cell count, ↓macrophages, ↓neutrophils, ↓Excessive proliferation of vascular smooth muscle cells | (Chen et al., 2021a) |
| **Blueberry Extract** |  | Polyphenols | Vaccinium myrtillus L. [Ericaceae] | In vivo | MCT-induced PAH model in SD rats | saline solution (0.9% NaCl) | In vivo: 100 mg/kg | 3 weeks | ↓ROS, ↓NADPH oxidase activity and lipid oxidation, ↑Nrf2, ↓ET-1. | (Turck et al., 2020; Leite et al., 2025) |
| **Safflower** |  | Flavonoids | Carthamus tinctorius L. | In vivo  In vitro | 1. MCT-induced PAH model in SD rats 2.PASMCs and PAECs from rats | saline | In vivo: 2 mL/kg  In vitro: 200 μg/mL | In vivo: 20 days  In vitro: 48 h | ↓NLRP3, **↓**TNF, ↓Th17, ↓IL-1β, ↓IL-18, ↓MMP-2, ↓MMP-9, ↓Collagen 1, ↓Collagen 3 | (Ding et al., 2024) |
| **Fermented cordyceps powder** |  | Fungal extract | Mycelium extracted from Cordyceps sinensis | In vivo | SD rats with HPH | sildenafil | In vivo: 0.4 g/kg | 28 days | ↓p38 MAPK/ NF-κB, ↓IL-1β,↓IL-6, ↓Pulmonary vascular inflammation, ↓abnormal proliferation of PASMC | (Li et al., 2023) |
| **Ethyl Acetate Extract of Sceptridium ternatum** |  | Flavones | Botrychium ternatum (Thunb.) Sw. [Ophioglossaceae] | In vivo | MCT-induced PAH model in SD rats | Bosentan | In vivo: 10 g/kg | 21 days | ↓NF-κB p65, ↓α-SMA, ↓pulmonary vascular remodeling | (Xin et al., 2020) |
| **tagitinin C** | C_19_H_24_O_6_ |  | Helianthus annuus L. [Asteraceae] | In vitro | PASMCs | HIF-1 inhibitor GN44028 | In vitro: 11.4 μM | 18 h | ↓HIF-1β/PDK1, ↓PDK1, ↓HIF-1β, ↓PDH, ↑TCA, ↓Warburg | (Arai et al., 2021) |
| **rutin** | C_27_H_30_O_16_ | Flavonoids | Ruta graveolens L. [Rutaceae] | In vivo  In vitro | 1. MCT-induced PAH rat model  2. Hypoxia-induced PASMCs | 0.3% sodium carboxymethyl cellulose | In vivo: 200 mg/kg  In vitro: 0.1 μmol/L | In vivo: 3 weeks  In vitro: 24 h | ↓PKCα, ↓PASMCs-Mitochondrial membrane rupture and reduced cristae. ↑FTH, ↑GPX4, ↓ACSL4, ↓Fe²⁺, ↓MDA, ↑GSH, ↓OS, ↓ferroptosis. | (Che et al., 2024) |
| **18β-glycyrrhetinic acid** | C_30_H_46_O_4_ | Triterpenoid aglycone | Glycyrrhiza glabra L. [Fabaceae] | In vivo | High-altitude pulmonary hypertension (HAPH) SD male rat model established by simulating high-altitude environment (5000 m) | Nifedipine | In vivo: 100 mg/kg | 15 days | ↑SOD, ↑GSH-Px, ↓MDA, ↑Glycolysis, ↑gluconeogenesis, ↑TCA. ↓BCAAs, ↑Proline, ↑Citrine, ↓Vascular fibrosis. ↓Ketone, ↓LDL | (Yang et al., 2021) |
| **kaempferol** | C_15_H_10_O_6_ | Flavonoid | Kaempferia galanga L. [Zingiberaceae]  Ginkgo biloba L. [Ginkgoaceae]  Moringa oleifera Lam. [Moringaceae] | In vivo  In vitro | 1. SD rat model of PAH induced by subcutaneous injection of MCT 2. Bovine PAECs under hypoxia | saline solution | In vivo: 150 mg/kg  In vitro: 30 μg/mL | In vivo: 3 weeks  In vitro: 24 h | ↓Succinic acid, ↓citric acid, ↓12-HETE, ↑DHA, ↓PTGS1 | (Yi et al., 2022) |
| **Osthole** | C_15_H_16_O_3_ | Coumarins | Angelica dahurica (Hoffm.) Benth. & Hook.f. ex Franch. & Sav. [Apiaceae]  Cnidium monnieri (L.) Cusson [Apiaceae] | In vivo  In vitro | 1. MCT-induced PAH rat model  2. PASMCs proliferation model induced with PDGF-BB | saline | In vivo: 80 mg/kg In vitro: 100 nM osthole | In vivo: 28 days  In vitro: 24 h | ↓microRNA-22-3p, ↓TC, ↓TG/HDL-C, ↓ CD36, ↓FAS, ↓Lipometabolism reprogramming | (Niu et al., 2022) |
| **chrysin** | C_15_H_10_O_4_ | Flavonoids | honey and propolis | In vivo | SU5416/hypoxia rat model | Su/Hx + vehicle (PBS) | In vivo: 100 mg/kg | 3 weeks | ↑Cd36, **↑**Cpt1b, ↓long-chain fatty acid, ↑Mitochondrial fatty acid oxidation, ↑Mitochondrial oxidative phosphorylation, ↑TCA | (Kobayashi et al., 2022) |
| **luteolin** | C_15_H_10_O_6_ | Flavonoids | Reseda odorata L. [Resedaceae] | In vivo  In vitro | 1. MCT-induced PAH rat model  2. PASMCs under hypoxia | saline | In vivo: 30 mg/kg  In vitro: 30 μmol/L | In vivo: 3 weeks  In vitro: 24 h | ↓ COX1, ↓5-LOX, ↓12-LOX, ↓15-LOX, ↓PGE2, ↓PGD2, ↓LTB4, ↓12-HETE, ↓15-HETE, ↓PGF2α, ↓8-iso-PGF2α, ↓ PASMC proliferation | (Song et al., 2022) |
| **L-citrulline** | C_6_H_13_N_3_O_3_ | Amino acids | Citrullus lanatus (Thunb.) Matsum. & Nakai [Cucurbitaceae] | In vivo | 1. Newborn pigs with chronic hypoxia-induced PH 2. Piglet PAECs | / | In vivo: 1.5 g/kg | 6-7 days | ↑ Arginine, ↑ eNOS substrate availability, ↑ eNOS enzyme coupling, ↓ Pulmonary artery superoxide generation, ↑ NO production, ↓ PVR, ↑ Vascular function | (Dikalova et al., 2020) |
| **Vanillic Acid** | C_8_H_8_O_4_ | Phenolic compound | Vanilla planifolia Andrews [Orchidaceae] | In vivo | MCT-induced PAH rat model | / | In vivo: 50 mg/kg and 100 mg/kg | 4 weeks | ↓ Arginase activity, ↓ Hif-2 α/Hif-1 β expression, ↑ eNOS, ↑ NO, ↓ Pulmonary vasoconstriction, ↓ Pulmonary vascular remodeling | (Wang et al., 2022) |
| **Qiliqiangxin capsule** |  |  | Astragalus mongholicus Bunge [Fabaceae]  Panax ginseng C.A.Mey. [Araliaceae]  Aconitum carmichaelii Debeaux [Ranunculaceae]  Salvia miltiorrhiza Bunge [Lamiaceae]  Descurainia sophia (L.) Webb ex Prantl [Brassicaceae]  Alisma plantago-aquatica subsp. orientale (Sam.) Sam. [Alismataceae]  Polygonatum odoratum (Mill.) Druce [Asparagaceae]  Neolitsea cassia (L.) Kosterm. [Lauraceae]  Carthamus tinctorius L. [Asteraceae]  Periploca sepium Bunge [Apocynaceae]  Citrus reticulata Blanco [Rutaceae] | In vivo | MCT-induced PAH SD rat model | saline | In vivo: 0.1 g/kg | 2 weeks | ↓ Glycolysis, ↓ Glucose uptake, ↑ Aerobic oxidation, ↑ PGC-1 α, ↑ Mitochondrial generation, ↑ Oxidative metabolism, ↑ Mitochondrial function, ↑ Oxidative phosphorylation, ↑ Oxidative phosphorylation | (Lu et al., 2020) |
| **green tea polyphenols** |  | Flavones | Camellia sinensis (L.) Kuntze [Theaceae] | In vitro | Hypoxia stimulated human PASMCs | / | In vitro: 10 μM | 24 h | ↓ AKT1 phosphorylation, ↓ HIF1 α, ↓ PDK1 generation, ↑ mitochondrial metabolism, ↓ HPASMCs proliferation and migration | (Yang et al., 2024) |
| **hawthorn flavonoid extract** |  | Flavonoids | Crataegus monogyna Jacq. [Rosaceae] | In vivo | High-altitude reared broiler chickens | 0 ml/L HFE of drinking water | In vivo: 0.2 ml/L | 42 dys | ↓ Oxygen free radicals, ↓ Oxygen free radicals, ↑ NO, ↓ Ca²⁺-ATPase, ↓ Intracellular Ca²⁺, ↓ Pulmonary arterial pressure | (Ahmadipour et al., 2020) |
| **Rhodiola crenulata extract** |  | Flavonoids and their glycosides | Rhodiola crenulata (Hook.f. & Thomson) H.Ohba [Crassulaceae] | In vivo | MCT-induced PAH rat model | saline | In vivo: 5 g/kg | 28 days | ↓ Acyl carnitine, ↓ CPT1A (mRNA/protein), ↓ FAO, reverse phospholipid metabolism imbalance, ↓ LC3B, ↓ ATG7, ↑ p62, ↓ autophagy, ↓ LKB1-AMPK | (Ren et al., 2021) |
| **Astragaloside IV** | C_41_H_68_O_14_ | Triterpenoid saponins | Astragalus mongholicus Bunge [Fabaceae] | In vivo  In vitro | 1. Chronic hypoxia-induced PH mice model 2. Mouse distal PASMCs 3. MCT-induced PAH SD rat model  4. Hypoxia-induced human PASMCs and PAECs | Sildenafil/ saline | In vivo: 80 mg/kg  In vitro: 40 μM  In vivo: 30 mg/kg  In vitro: ① 80 μM, ② 20 μM | In vivo: 3 weeks/4 weeks  In vitro: 72 h/24 h | ↓ mTOR phosphorylation, ↓ Bcl-6 (Tfh), ↓ Bcl-6 (Tfh), reshaping Tfh/Tfr balance, ↓ IL-21, ↓ NF - κ B, ↓ inflammatory response, ↑ PHD2, ↓ HIF1 α, ↓ HIF1 α/NLRP3, ↓ PASMCs pyroptosis, ↓ Collagen I. ↓ mTOR/RhoA, ↑ p27, ↓ PASMCs proliferation and migration | (Jin et al., 2021; Li et al., 2022; XinTian et al., 2022; Xi et al., 2023) |
| **Quercetin** | C_15_H_10_O_7_ | Flavonoids | Coptis chinensis Franch. [Ranunculaceae] | In vivo  In vitro | 1. SD male rat with hypoxic PAH 2. MCT-induced PAH SD rat model  3. PASMCs and PAECs treated with MCT | Saline/ 0.5% carboxymethylcellulose sodium salt | In vivo: ① 30 mg/kg, ② 50 mg/kg In vitro: 5 μm and 2.5 μm | In vivo: 4 weeks/3 weeks  In vitro: 24 h | ↓HMGB1/RAGE/NF-κB, ↓TNF-α、IL-6, ↓NLRP3, ↓IL-1β/IL-18 maturation, ↑miR-204, ↓HIF1α/NFATc2, ↓Src-STAT3-NFAT, restore the balance of immune cell apoptosis.↓CYP1A1, ↓UGT1A9, ↓MAPK1（ERK1/2）phosphorylation, ↓NOX4, ↓ROS, ↓TGF-β1, NF-κB ↔ NOX4. | (Luo et al., 2021; Rajabi et al., 2021; Zhang et al., 2023a; Ding et al., 2024; Kordestani et al., 2024; Li et al., 2024) |
| **Berberine** | C_20_H_18_NO_4_^+^ | Alkaloids | Phellodendron amurense Rupr. [Rutaceae]  Coptis chinensis Franch. [Ranunculaceae] | In vivo  In vitro | 1. MCT-induced PAH SD rat model  2. Hypoxia-induced PASMCs from SD rats 3. SU5416/hypoxia-induced PAH rats 4. Human PASMCs, exposed to hypoxia | saline | In vivo: ① 50 mg/kg, ② 30 mg/kg In vitro: 5 μm In vivo: 100 mg/kg In vitro: 10 μmol/L | In vivo: 4 weeks/3 weeks  In vitro: 24 h | ↓Trx1, ↓β-catenin nuclear translocation, ↓cyclin D1、VEGF, ↓MAPK1（ERK1/2）phosphorylation, Blocking metabolic reprogramming of PASMCs. ↓NOX4, ↓ROS. ↓CYP1B1, ↓NF-κB, ↓TNF-α、IL-6, ↑miR-204, ↓HIF1α/NFATc2, ↓TGF-β1, ↑Bax, ↓Bcl-2. | (Yu et al., 2020; Luo et al., 2021; Rajabi et al., 2021; Kordestani et al., 2024) |
| **resveratrol** | C_14_H_12_O_3_ | Polyphenols | Veratrum album L. [Melanthiaceae] | In vivo  In vitro | 1. SD rats with hypoxia-induced PH 2. SD rat with CTEPH induced by repeated injection of autologous blood clots into the left jugular vein 3. MCT-induced PAH SD rat model  4. Human PAECs stimulated with thrombin | saline | In vivo: ① 40 mg/kg, ② 10 mg/kg, ③ 20 mg/kg In vitro: 10 μm | In vivo: 3 weeks/4 weeks/42 days  In vitro: 24 h | ↓ STAT3 phosphorylation, ↓ Th17 differentiation, ↓ IL-6, ↓VCAM-1, ↑ Nrf2/Trx-1, ↑ LC3-II, ↓ vWF/P-selectin, ↓ TGF-β/Smad3, ↑ TCA, ↑ SIRT1, ↓ Pulmonary vascular remodeling | (Li et al., 2020; Vazquez-Garza et al., 2020; Sun et al., 2021; Liu et al., 2022) |
| **Scutellarein** | C_15_H_10_O_6_ | Flavonoids | Scutellaria baicalensis Georgi [Lamiaceae]  Erigeron breviscapus (Vaniot) Hand.-Mazz. [Asteraceae] | In vivo  In vitro | 1. MCT-induced PAH rat model 2. Hypoxia + Su5416 (HxSu)-induced PAH mouse model 3. Human PASMCs | / | In vivo: 50 mg/kg  In vitro: 50 μm | In vivo: 3 weeks/4 weeks  In vitro: 48 h | ↑SIRT1, ↑NNT deacetylation, ↑NAD⁺, Maintain mitochondrial metabolic homeostasis. ↓NLRP3, ↓immune inflammation. | (Tang et al., 2025) |
| **ginsenoside Rg1** | C_42_H_72_O_14_ | Ginsenosides | Panax ginseng C.A.Mey. [Araliaceae] | In vivo  In vitro | 1. C57BL/6 mice and calpain-1 knockout mice exposed to 10% oxygen 2. PASMCs from SD rats cultured under 3% oxygen condition 3. Hypoxia combined with su5416 (VEGFR inhibitor)-induced PAH mouse model 4. Hypoxia-induced human PAECs | Sildenafil | In vivo: 20 mg/kg In vitro: 20 μm | In vivo: 4 weeks  In vitro: 24 h | ↓calpain-1, ↓STAT3 phosphorylation, ↓IL-6/STAT3, ↓TXNIP/NLRP3, ↓IL-1β, ↓Mitochondrial OS, ↓PINK1/Parkin, ↑antioxidant enzyme activity, ↑eNOS/NO, Improve endothelial function. | (Ran et al., 2024; Zhang et al., 2025) |
| **2-phenylethyl-beta-glucopyranoside** | C_14_H_20_O_6_ |  | Rehmannia glutinosa (Gaertn.) Libosch. ex DC. [Orobanchaceae] | In vivo  In vitro | 1. Hypoxia-induced SPF male C57BL/6N mice 2. Hypoxia-induced PASMCs | bosentan | In vivo: 40 mg/kg In vitro: 10 μmol/L | In vivo: 14 days  In vitro: 48 h | ↓PI3K/Akt/mTOR, ↓ROS, ↓MDA, ↑SOD, ↑GSH-Px, ↓OS. ↓MDSCs, ↓Tregs, ↑NK. | (Zeng et al., 2024) |
| **Hydroxysafflor yellow A** | C_27_H_32_O_16_ |  | Carthamus tinctorius L. [Asteraceae] | In vivo | MCT-induced PAH rat model | saline | In vivo: 220 mg/kg | In vivo: 4 weeks | ↑ANXA5, ↑SRC, ↑PPARG, ↑lipid metabolism, ↓OS. | (Ji et al., 2024) |
| **Lingguizhugan decoction** |  |  | Poria cocos(Schw.)Wolf  Neolitsea cassia (L.) Kosterm. [Lauraceae]  Atractylodes macrocephala Koidz. [Asteraceae]  Glycyrrhiza glabra L. [Fabaceae] | In vivo |  | Captopril | In vivo: 6.3 g/kg and 12.6 g/kg | 28 days | ↓CCL2/CXCR4, ↓M2, ↓HMOX1/NOX4, ↓Iron death, regulation of PPAR and fatty acid metabolism | (Shi et al., 2024) |
| **Zhishi-Xiebai-Guizhi Decoction** |  |  | Trichosanthes kirilowii Maxim. [Cucurbitaceae] Allium chinense G.Don [Amaryllidaceae] Citrus × aurantium f. aurantium [Rutaceae] Magnolia officinalis Rehder & E.H.Wilson [Magnoliaceae] Neolitsea cassia (L.) Kosterm. [Lauraceae] | In vivo  In vitro | 1. Hypoxic HPH rat model  2. MCT-induced PAH rat model  3. PDGF-BB-stimulated PASMCs | Saline/Sildenafil | In vivo: ① 880 mg/kg ② 2.7 g/kg In vitro: 10 mg/mL | In vivo: 4 weeks/14 days  In vitro: 24 h | ↓IL-6, ↓TNF, ↓HIF-1α/PI3K/Akt, ↓Warburg Effect, → IL-6/IL-10/PLIN2 | (Fu et al., 2024; Huang et al., 2024) |
| **Shufeiya Recipe** |  |  | Salvia miltiorrhiza Bunge [Lamiaceae] Carthamus tinctorius L. [Asteraceae] Cornus officinalis Siebold & Zucc. [Cornaceae] Platycodon grandiflorus (Jacq.) A.DC. [Campanulaceae] | In vivo | MCT-induced PAH rat model | Sildenafil | In vivo: 1.1700 g/mL | 14 days | ↑SIRT3/FOXO3a, ↑Mn-SOD, ↑COX-1/2, ↓OS. ↑PI3K/AKT/eNOS, ↑NO/sGC/cGMP/PKG, ↓Ras/MEK1/2/ERK1/2/c-fos. | (Jia et al., 2022) |
| **Qishen Yiqi Formula** |  |  | Astragalus mongholicus Bunge [Fabaceae] Salvia miltiorrhiza Bunge [Lamiaceae] Panax notoginseng (Burkill) F.H.Chen [Araliaceae] Dalbergia odorifera T.C.Chen [Fabaceae] |  |  |  |  |  | ↑TP53, ↓NF-κB, HIF-1, ↓PASMCs Metabolic reprogramming | (Wu et al., 2021) |
| **extract of Salvia przewalskii** |  |  | Salvia przewalskii Maxim. [Lamiaceae] | In vivo | 1. Kunming mice with acute hypoxia model 2. SD rats with PAH induced by low pressure and chronic hypoxia | ACTZ/sildenafil/ Saline | In vivo: ① 1.0 g/kg ② 2.0 g/kg | 7 days/4 weeks | ↓HIF-1α, ↓PCNA, ↓HIF-1α, ↓PCNA, ↓Bcl-2, ↓CDK4, ↓CyclinD1, ↓P27Kip1, ↓ RhoA-ROCK, ↓ MCP-1, ↓ NF-κB, ↓abnormal cell proliferation and vascular remodeling. ↑SOD, ↑LDH, ↓MDA, ↓OS. | (Wang et al., 2020) |
| **Grape seed procyanidin** |  | Polyphenolic flavonoid combination | Vitis vinifera L. [Vitaceae] | In vivo  In vitro | 1. Cigarette smoke-exposed SD rat 2. CSE-treated rat primary PASMCs | Saline | In vivo: 30 mg/kg  In vitro: 4 g/L | In vivo: 6 months  In vitro: 24 h | ↑PPARγ, ↓COX-2, regulating lipid metabolism, ↓ cell proliferation, ↓ inflammatory response, ↓ vascular remodeling | (Liu et al., 2020) |
| **Aureane-type sesquiterpene tetraketides** |  | Aureane-type sesquiterpene tetraketides | Wetland mud-derived fungus, Myrothecium gramineum | In vivo | 1. Hypoxia-induced PH mouse model 2. MCT-induced PH rat model | Saline | In vivo: 20 mg/kg | 4 weeks | ↓RORγt, ↓IL-17A, ↓Warburg Effect, ↓Th17, ↓Inflammatory response, ↓PASMC proliferation | (Tang et al., 2023) |

**Reference**:

Ahmadipour, B., Kalantar, M., Schreurs, N.M., Raza, S.H.A., Khan, R., Khan, S., et al. (2020). Flavonoid bioactive compounds of hawthorn extract can promote growth, regulate electrocardiogram waves, and improve cardiac parameters of pulmonary hypertensive chickens. *POULTRY SCIENCE* 99(2)**,** 974-980. doi: 10.1016/j.psj.2019.10.022.

Arai, M.A., Sakuraba, K., Makita, Y., Hara, Y., and Ishibashi, M. (2021). Evaluation of Naturally Occurring HIF-1 Inhibitors for Pulmonary Arterial Hypertension. *CHEMBIOCHEM* 22(18)**,** 2799-2804. doi: 10.1002/cbic.202100223.

Chang, Z., Wang, J.-l., Jing, Z.-c., Ma, P., Xu, Q.-b., Na, J.-r., et al. (2020). Protective effects of isorhamnetin on pulmonary arterial hypertension: in vivo and in vitro studies. *PHYTOTHERAPY RESEARCH* 34(10)**,** 2730-2744. doi: 10.1002/ptr.6714.

Che, H., Yi, J., Zhao, X., Yu, H., Wang, X., Zhang, R., et al. (2024). Characterization of PKCα-rutin interactions and their application as a treatment strategy for pulmonary arterial hypertension by inhibiting ferroptosis. *FOOD & FUNCTION* 15(2)**,** 779-793. doi: 10.1039/d3fo01306e.

Chen, L., Ren, L., Song, H., Ren, H., Xu, R., and Huang, X. (2021a). Triflavones from Selaginella Doederllein Inhibited Hypoxia-induced, Pulmonary Vascular Remodeling Through PI3K/Akt. *ALTERNATIVE THERAPIES IN HEALTH AND MEDICINE* 27(6)**,** 34-39.

Chen, Y., Cui, L., Wang, C., Liu, J., and Guo, J. (2021b). Ameliorative Effects and Mechanism of Buyang Huanwu Decoction on Pulmonary Vascular Remodeling: Network and Experimental Analyses. *OXIDATIVE MEDICINE AND CELLULAR LONGEVITY* 2021. doi: 10.1155/2021/4576071.

Dikalova, A., Aschner, J.L., Kaplowitz, M.R., Cunningham, G., Summar, M., and Fike, C.D. (2020). Combined L-citrulline and tetrahydrobiopterin therapy improves NO signaling and ameliorates chronic hypoxia-induced pulmonary hypertension in newborn pigs. *AMERICAN JOURNAL OF PHYSIOLOGY-LUNG CELLULAR AND MOLECULAR PHYSIOLOGY* 318(4)**,** L762-L772. doi: 10.1152/ajplung.00280.2019.

Ding, S., Cui, J., Yan, L., Ru, C., He, F., and Chen, A. (2024). Safflower Alleviates Pulmonary Arterial Hypertension by Inactivating NLRP3: A Combined Approach of Network Pharmacology and Experimental Verification. *CLINICAL RESPIRATORY JOURNAL* 18(8). doi: 10.1111/crj.13826.

Fu, M., Li, Y., Liu, J., Liu, J., Wei, J., Qiao, Y., et al. (2024). Zhishi Xiebai Guizhi Decoction modulates hypoxia and lipid toxicity to alleviate pulmonary vascular remodeling of pulmonary hypertension in rats. *CHINESE MEDICINE* 19(1). doi: 10.1186/s13020-024-01039-0.

Gao, A.-R., Li, S., Tan, X.-C., Huang, T., Dong, H.-J., Xue, R., et al. (2022). Xinyang Tablet attenuates chronic hypoxia-induced right ventricular remodeling via inhibiting cardiomyocytes apoptosis. *CHINESE MEDICINE* 17(1). doi: 10.1186/s13020-022-00689-2.

Huang, L., Li, H., Huang, S., Wang, S., Liu, Q., Luo, L., et al. (2022). Notopterol Attenuates Monocrotaline-Induced Pulmonary Arterial Hypertension in Rat. *FRONTIERS IN CARDIOVASCULAR MEDICINE* 9. doi: 10.3389/fcvm.2022.859422.

Huang, P., Wang, Y., Liu, C., Zhang, Q., Ma, Y., Liu, H., et al. (2024). Exploring the Mechanism of Zhishi-Xiebai-Guizhi Decoction for the Treatment of Hypoxic Pulmonary Hypertension based on Network Pharmacology and Experimental Analyses. *CURRENT PHARMACEUTICAL DESIGN* 30(26)**,** 2059-2074. doi: 10.2174/0113816128293601240523063527.

Ji, X.Y., Lei, C.-J., Kong, S., Li, H.-F., Chen, Y.-J., Zhao, F.-R., et al. (2024). Hydroxy-Safflower Yellow A Mitigates Vascular Remodeling in Rat Pulmonary Arterial Hypertension. *DRUG DESIGN DEVELOPMENT AND THERAPY* 18**,** 475-491. doi: 10.2147/DDDT.S439686.

Jia, Z., Yan, H., Wang, S., Wang, L., Cao, Y., Lin, S., et al. (2022). Shufeiya Recipe Improves Monocrotaline-Induced Pulmonary Hypertension in Rats by Regulating SIRT3/FOXO3a and Its Downstream Signaling Pathways. *DISEASE MARKERS* 2022. doi: 10.1155/2022/3229888.

Jin, H., Jiao, Y., Guo, L., Ma, Y., Zhao, R., Li, X., et al. (2021). Astragaloside IV blocks monocrotaline-induced pulmonary arterial hypertension by improving inflammation and pulmonary artery remodeling. *INTERNATIONAL JOURNAL OF MOLECULAR MEDICINE* 47(2)**,** 595-606. doi: 10.3892/ijmm.2020.4813.

Kobayashi, T., Kim, J.-D., Naito, A., Yanagisawa, A., Jujo-Sanada, T., Kasuya, Y., et al. (2022). Multi-omics analysis of right ventricles in rat models of pulmonary arterial hypertension: Consideration of mitochondrial biogenesis by chrysin. *INTERNATIONAL JOURNAL OF MOLECULAR MEDICINE* 49(5). doi: 10.3892/ijmm.2022.5124.

Kordestani, Z., Beik, A., Najafipour, H., Safi, Z., Askaripour, M., and Rajabi, S. (2024). Perillyl alcohol, quercetin, and berberine combination therapy ameliorates experimental pulmonary arterial hypertension: Effects on the lung miR-204 expression, remodeling, and inflammatory factors. *Avicenna journal of phytomedicine* 14(6)**,** 764-775. doi: 10.22038/AJP.2024.24522.

Leite, L.B., Soares, L.L., Guimaraes-Ervilha, L.O., Costa, S.F.F., Generoso, S.C.d.L., Xavier, M.A.M., et al. (2025). Blueberry Extract and Resistance Training Prevent Left Ventricular Redox Dysregulation and Pathological Remodeling in Experimental Severe Pulmonary Arterial Hypertension. *NUTRIENTS* 17(7). doi: 10.3390/nu17071145.

Li, C., Peng, G., Long, J., Xiao, P., Zeng, X., and Yang, H. (2020). Protective effects of resveratrol and SR1001 on hypoxia-induced pulmonary hypertension in rats. *CLINICAL AND EXPERIMENTAL HYPERTENSION* 42(6)**,** 519-526. doi: 10.1080/10641963.2020.1714643.

Li, C., Zhu, H., Zhang, S., Meng, F., Li, S., Li, G., et al. (2022). Astragaloside IV ameliorates pulmonary vascular remodeling in hypoxia-induced pulmonary hypertension by restraining the T follicular helper cell response and expanding T follicular regulatory cell response. *PHYTOMEDICINE* 102. doi: 10.1016/j.phymed.2022.154171.

Li, J., Chen, T., Yang, Z., Su, S., Wang, Y., Li, Z., et al. (2023). Fermented Cordyceps Powder Attenuates Inflammation and Pulmonary Arterioles Remodeling by Inhibiting p38 MAPK/NF-κB Signaling Pathway in Hypoxic Pulmonary Hypertension Rats. *PHARMACOGNOSY MAGAZINE* 19(4)**,** 841-852. doi: 10.1177/09731296231194402.

Li, Q., Chen, X., Zhang, S., Li, W., and Lin, H. (2024). Analysis of riociguat and desmethyl riociguat by UPLC-MS/MS and its interaction with quercetin. *FRONTIERS IN PHARMACOLOGY* 15. doi: 10.3389/fphar.2024.1470377.

Li, Q., and Zhang, H. (2023). Bioinformatics analysis to identify potential biomarkers for the pulmonary artery hypertension associated with the basement membrane. *OPEN LIFE SCIENCES* 18(1). doi: 10.1515/biol-2022-0730.

Liu, J., Hu, S., Zhu, B., Shao, S., and Yuan, L. (2020). Grape seed procyanidin suppresses inflammation in cigarette smoke-exposed pulmonary arterial hypertension rats by the PPAR-γ/COX-2 pathway. *NUTRITION METABOLISM AND CARDIOVASCULAR DISEASES* 30(2)**,** 347-354. doi: 10.1016/j.numecd.2019.09.022.

Liu, X., Zhou, H., and Hu, Z. (2022). Resveratrol attenuates chronic pulmonary embolism-related endothelial cell injury by modulating oxidative stress, inflammation, and autophagy. *CLINICS* 77. doi: 10.1016/j.clinsp.2022.100083.

Lu, Y., Wu, J., Sun, Y., Xin, L., Jiang, Z., Lin, H., et al. (2020). Qiliqiangxin prevents right ventricular remodeling by inhibiting apoptosis and improving metabolism reprogramming with pulmonary arterial hypertension. *AMERICAN JOURNAL OF TRANSLATIONAL RESEARCH* 12(9)**,** 5655-5669.

Luo, S., Kan, J., Zhang, J., Ye, P., Wang, D., Jiang, X., et al. (2021). Bioactive Compounds From Coptidis Rhizoma Alleviate Pulmonary Arterial Hypertension by Inhibiting Pulmonary Artery Smooth Muscle Cells' Proliferation and Migration. *JOURNAL OF CARDIOVASCULAR PHARMACOLOGY* 78(2)**,** 253-262. doi: 10.1097/FJC.0000000000001068.

Ma, Q., Wang, M., Li, L., Zhang, X., Cui, L., Mou, J., et al. (2023). Jiedu Quyu Decoction mitigates monocrotaline-induced right-sided heart failure associated with pulmonary artery hypertension by inhibiting NLRP3 inflammasome in rats. *JOURNAL OF ETHNOPHARMACOLOGY* 313. doi: 10.1016/j.jep.2023.116556.

Niu, Z., Fu, M., Li, Y., Ren, H., Zhang, X., and Yao, L. (2022). Osthole alleviates pulmonary vascular remodeling by modulating microRNA-22-3p mediated lipid metabolic reprogramming. *PHYTOMEDICINE* 96. doi: 10.1016/j.phymed.2021.153840.

Rajabi, S., Najafipour, H., Farsangi, S.J., Joukar, S., Beik, A., Iranpour, M., et al. (2020). Perillyle alcohol and Quercetin ameliorate monocrotaline-induced pulmonary artery hypertension in rats through PARP1-mediated miR-204 down-regulation and its downstream pathway. *BMC COMPLEMENTARY MEDICINE AND THERAPIES* 20(1). doi: 10.1186/s12906-020-03015-1.

Rajabi, S., Najafipour, H., Jafarinejad-Farsangi, S., Joukar, S., Beik, A., Askaripour, M., et al. (2021). Quercetin, Perillyl Alcohol, and Berberine Ameliorate Right Ventricular Disorders in Experimental Pulmonary Arterial Hypertension: Effects on miR-204, miR-27a, Fibrotic, Apoptotic, and Inflammatory Factors. *JOURNAL OF CARDIOVASCULAR PHARMACOLOGY* 77(6)**,** 777-786. doi: 10.1097/FJC.0000000000001015.

Ran, C., Lu, M., Zhao, F., Hao, Y., Guo, X., Li, Y., et al. (2024). Ginsenoside Rg1 alleviates vascular remodeling in hypoxia-induced pulmonary hypertension mice through the calpain-1/STAT3 signaling pathway. *JOURNAL OF GINSENG RESEARCH* 48(4)**,** 405-416. doi: 10.1016/j.jgr.2024.03.001.

Ren, H.-H., Niu, Z., Guo, R., Fu, M., Li, H.-R., Zhang, X.-Y., et al. (2021). Rhodiola crenulata extract decreases fatty acid oxidation and autophagy to ameliorate pulmonary arterial hypertension by targeting inhibiton of acylcarnitine in rats. *CHINESE JOURNAL OF NATURAL MEDICINES* 19(2)**,** 120-133. doi: 10.1016/S1875-5364(21)60013-4.

Shi, S., Fu, Z., Wang, Y., Duan, C., Hu, S., Wu, H., et al. (2024). Exploring the inflammation-related mechanisms of Lingguizhugan decoction on right ventricular remodeling secondary to pulmonary arterial hypertension based on integrated strategy using UPLC-HRMS, systems biology approach, and experimental validation. *PHYTOMEDICINE* 132. doi: 10.1016/j.phymed.2024.155879.

Song, K., Duan, Q., Ren, J., Yi, J., Yu, H., Che, H., et al. (2022). Targeted metabolomics combined with network pharmacology to reveal the protective role of luteolin in pulmonary arterial hypertension. *FOOD & FUNCTION* 13(20)**,** 10695-10709. doi: 10.1039/d2fo01424f.

Sun, X., Zhao, B., Qu, H., Chen, S., Hao, X., Chen, S., et al. (2021). Sera and lungs metabonomics reveals key metabolites of resveratrol protecting against PAH in rats. *BIOMEDICINE & PHARMACOTHERAPY* 133. doi: 10.1016/j.biopha.2020.110910.

Tang, H., Ning, K., Wu, B., Wang, X., He, J., Li, P., et al. (2025). Scutellarein ameliorates pulmonary arterial hypertension via sirtuin 1 mediated deacetylation of nicotinamide nucleotide transhydrogenase. *BIOCHEMICAL PHARMACOLOGY* 237. doi: 10.1016/j.bcp.2025.116932.

Tang, X., Wang, C., Wang, L., Ren, F., Kuang, R., Li, Z., et al. (2023). Aureane-type sesquiterpene tetraketides as a novel class of immunomodulators with interleukin-17A inhibitory activity. *ACTA PHARMACEUTICA SINICA B* 13(9)**,** 3930-3944. doi: 10.1016/j.apsb.2023.03.017.

Turck, P., Fraga, S., Salvador, I., Campos-Carraro, C., Lacerda, D., Bahr, A., et al. (2020). Blueberry extract decreases oxidative stress and improves functional parameters in lungs from rats with pulmonary arterial hypertension. *NUTRITION* 70. doi: 10.1016/j.nut.2019.110579.

Vazquez-Garza, E., Bernal-Ramirez, J., Jerjes-Sanchez, C., Lozano, O., Acuna-Morin, E., Vanoye-Tamez, M., et al. (2020). Resveratrol Prevents Right Ventricle Remodeling and Dysfunction in Monocrotaline-Induced Pulmonary Arterial Hypertension with a Limited Improvement in the Lung Vasculature. *OXIDATIVE MEDICINE AND CELLULAR LONGEVITY* 2020. doi: 10.1155/2020/1841527.

Wang, J., Guan, L., Yu, J., Ma, B., Shen, H., Xing, G., et al. (2025). Halofuginone prevents inflammation and proliferation of high-altitude pulmonary hypertension by inhibiting the TGF-β1/Smad signaling pathway. *SCIENTIFIC REPORTS* 15(1). doi: 10.1038/s41598-025-88258-z.

Wang, S., Sun, X., Wang, Z., Zhou, S., Su, S., Nan, X., et al. (2022). Vanillic Acid Attenuates Monocrotaline-Induced Pulmonary Arterial Hypertension by Enhancing NO Signaling Pathways. *NATURAL PRODUCT COMMUNICATIONS* 17(9). doi: 10.1177/1934578X221128411.

Wang, Y., Duo, D., Yan, Y., He, R., Wang, S., Wang, A., et al. (2020). Extract of Salvia przewalskii Repair Tissue Damage in Chronic Hypoxia Maybe through the RhoA-ROCK Signalling Pathway. *BIOLOGICAL & PHARMACEUTICAL BULLETIN* 43(3)**,** 432-439.

Wu, P., Xie, X., Chen, M., Sun, J., Cai, L., Wei, J., et al. (2021). Elucidation of the Mechanisms and Molecular Targets of Qishen Yiqi Formula for the Treatment of Pulmonary Arterial Hypertension using a Bioinformatics/Network Topology-based Strategy. *COMBINATORIAL CHEMISTRY & HIGH THROUGHPUT SCREENING* 24(5)**,** 701-715. doi: 10.2174/1386207323666201019145354.

Xi, J., Ma, Y., Liu, D., and Li, R. (2023). Astragaloside IV restrains pyroptosis and fibrotic development of pulmonary artery smooth muscle cells to ameliorate pulmonary artery hypertension through the PHD2/HIF1α signaling pathway. *BMC PULMONARY MEDICINE* 23(1). doi: 10.1186/s12890-023-02660-9.

Xin, W.-x., Li, Q.-l., Fang, L., Zhong, L.-k., Zheng, X.-w., and Huang, P. (2020). Preventive Effect and Mechanism of Ethyl Acetate Extract of Sceptridium ternatum in Monocrotaline-Induced Pulmonary Arterial Hypertension. *CHINESE JOURNAL OF INTEGRATIVE MEDICINE* 26(3)**,** 205-211. doi: 10.1007/s11655-018-2573-6.

XinTian, Zhang, X., Feng, Y., Gao, X., Hao, X., Zhang, J., et al. (2022). Astragaloside IV in Hypoxic Pulmonary Hypertension: an In Vivo and In Vitro Experiments. *APPLIED BIOCHEMISTRY AND BIOTECHNOLOGY* 194(12)**,** 6319-6334. doi: 10.1007/s12010-022-04027-y.

Xue, X., Zhang, S., Jiang, W., Wang, J., Xin, Q., Sun, C., et al. (2021). Protective effect of baicalin against pulmonary arterial hypertension vascular remodeling through regulation of TNF-α signaling pathway. *PHARMACOLOGY RESEARCH & PERSPECTIVES* 9(1). doi: 10.1002/prp2.703.

Yang, H., Cao, J., Li, J.-M., Li, C., Zhou, W.-W., and Luo, J.-W. (2024). Exploration of the molecular mechanism of tea polyphenols against pulmonary hypertension by integrative approach of network pharmacology, molecular docking, and experimental verification. *MOLECULAR DIVERSITY* 28(4)**,** 2603-2616. doi: 10.1007/s11030-023-10700-z.

Yang, T., Zhou, J., Fang, L., Wang, M., Dilinuer, M., and Ainiwaer, A. (2021). Protection function of 18β-glycyrrhetinic acid on rats with high-altitude pulmonary hypertension based on 1H NMR metabonomics technology. *ANALYTICAL BIOCHEMISTRY* 631. doi: 10.1016/j.ab.2021.114342.

Yi, J., Wang, X., Song, K., Ren, J., Che, H., Yu, H., et al. (2022). Integrated metabolomics and mechanism to reveal the protective effect of kaempferol on pulmonary arterial hypertension. *JOURNAL OF PHARMACEUTICAL AND BIOMEDICAL ANALYSIS* 212. doi: 10.1016/j.jpba.2022.114662.

Yu, W., Luo, J., Zhang, A., Zheng, Y., Zhu, L., Gu, Y., et al. (2020). Berberine alleviates pulmonary hypertension through Trx1 and β-catenin signaling pathways in pulmonary artery smooth muscle cells. *EXPERIMENTAL CELL RESEARCH* 390(1). doi: 10.1016/j.yexcr.2020.111910.

Zeng, M.-N., Zhang, Y.-H., Guo, P.-L., Zhang, Z.-Y., Liu, Y.-L., Wang, R., et al. (2024). [Effect of 2-phenylethyl-beta-glucopyranoside isolated from Huaizhong No. 1 Rehmannia glutinosa on hypoxic pulmonary hypertension by regulating PI3K/Akt/m TOR/HIF-1alpha pathway]. *Zhongguo Zhong yao za zhi = Zhongguo zhongyao zazhi = China journal of Chinese materia medica* 49(14)**,** 3857-3867. doi: 10.19540/j.cnki.cjcmm.20240412.701.

Zhang, N., Qiu, Q., Chen, Y., Sun, Z., Lu, G., Wang, L., et al. (2023a). [Quercetin improves pulmonary arterial hypertension in rats by regulating the HMGB1/RAGE/NF-kappaB pathway]. *Nan fang yi ke da xue xue bao = Journal of Southern Medical University* 43(9)**,** 1606-1612. doi: 10.12122/j.issn.1673-4254.2023.09.19.

Zhang, Q., Chen, Y., Wang, Q., Wang, Y., Feng, W., Chai, L., et al. (2023b). HMGB1-induced activation of ER stress contributes to pulmonary artery hypertension in vitro and in vivo. *RESPIRATORY RESEARCH* 24(1). doi: 10.1186/s12931-023-02454-x.

Zhang, R., Lu, M., Ran, C., Niu, L., Qi, Q., and Wang, H. (2025). Ginsenoside Rg1 improves hypoxia-induced pulmonary vascular endothelial dysfunction through TXNIP/NLRP3 pathway-modulated mitophagy. *JOURNAL OF GINSENG RESEARCH* 49(1)**,** 80-91. doi: 10.1016/j.jgr.2024.10.002.

Zhu, Y., Sun, Y., Zhang, S., Li, C., Zhao, Y., Zhao, B., et al. (2021). Xinmai 'an extract enhances the efficacy of sildenafil in the treatment of pulmonary arterial hypertension via inhibiting MAPK signalling pathway. *PHARMACEUTICAL BIOLOGY* 59(1)**,** 594-605. doi: 10.1080/13880209.2021.1917629.
